# Insights into the Divergence of Chinese *Ips* Bark Beetles during Evolutionary Adaptation

**DOI:** 10.3390/biology11030384

**Published:** 2022-02-28

**Authors:** Huicong Du, Jiaxing Fang, Xia Shi, Chunmei Yu, Mei Deng, Sufang Zhang, Fu Liu, Zhen Zhang, Fuzhong Han, Xiangbo Kong

**Affiliations:** 1 Key Laboratory of Forest Protection of National Forestry and Grassland Administration, Institute of Forest Ecology, Environment and Nature Conservation, Chinese Academy of Forestry, Beijing 100091, China; dhc962@caf.ac.cn (H.D.); fjxinsect@163.com (J.F.); m18304018586@163.com (X.S.); zhangsf@caf.ac.cn (S.Z.); liufu2006@163.com (F.L.); zhangzhen@caf.ac.cn (Z.Z.); 2 Forest Diseases and Pest Control and Quarantine General Station of Qinghai Province, Xining 810007, China; qxshu@126.com (C.Y.); deimei1975@126.com (M.D.); 3 Maixiu Forest Farm, Huangnan 811399, China; hanfz001@126.com

**Keywords:** bark beetle, mitochondrial genome, genetic distance, evolutionary path, aggregation pheromone

## Abstract

**Simple Summary:**

Bark beetle species of the genus *Ips* are among the major pests of Chinese conifer forests. Based on mitochondrial genome and SNP, we investigated the phylogenetic relationships and evolutionary trends of 19 populations of six *Ips* species that had serious outbreaks in recent years. Our results demonstrated the relationships between *Ips* evolution and host plants, pheromones, and altitudinal differences, and provided new insights into the mechanism of adaptive evolution of *Ips* bark beetles.

**Abstract:**

Many bark beetles of the genus *Ips* are economically important insect pests that cause severe damage to conifer forests worldwide. In this study, sequencing the mitochondrial genome and restriction site-associated DNA of *Ips* bark beetles helps us understand their phylogenetic relationships, biogeographic history, and evolution of ecological traits (e.g., pheromones and host plants). Our results show that the same topology in phylogenetic trees constructed in different ways (ML/MP/BI) and with different data (mtDNA/SNP) helps us to clarify the phylogenetic relationships between Chinese *Ips* bark beetle populations and Euramerican species and their higher order clades; *Ips* bark beetles are polyphyletic. The structure of the mitochondrial genome of *Ips* bark beetles is similar and conserved to some extent, especially in the sibling species *Ips typographus* and *Ips nitidus*. Genetic differences among *Ips* species are mainly related to their geographic distribution and different hosts. The evolutionary pattern of aggregation pheromones of *Ips* species reflects their adaptations to the environment and differences among hosts in their evolutionary process. The evolution of *Ips* species is closely related to the uplift of the Qinghai-Tibet Plateau and host switching. Our study addresses the evolutionary trend and phylogenetic relationships of *Ips* bark beetles in China, and also provides a new perspective on the evolution of bark beetles and their relationships with host plants and pheromones.

## 1. Introduction

Tree-destroying bark beetles (Coleoptera, Curculionidae, Scolytinae) are the most destructive pests of conifer forests worldwide and, therefore, are of considerable ecological importance [1]. The diversity of factors that determine the population dynamics of bark beetles in forest ecosystems is a challenge for many researchers. These include host phytochemistry [2,3], host range expansion [4], beetle–microbe interactions [5,6], influences of natural enemies, climatic changes, and microhabitat differences [7,8]. Bark beetles of the genus *Ips* are mainly distributed in conifer forests of the Northern Hemisphere. To date, 43 species of the genus *Ips* have been taxonomically identified worldwide, of which 27 species occur only in North and Central America and 16 species are distributed in Eurasia [9], including some of the most ecologically and economically important native species in China. 

The phylogenetic relationships of some *Ips* species/populations in Europe and North America have been studied [10,11,12]. For example, Stauffer et al. [13] investigated the phylogenetic relationships of seven European *Ips* species based on *COI* for the first time, and Cognato also investigated the phylogenetic relationships of 39 *Ips* species from Europe and North America and suggested that *Ips* species diversity in Asia may be underestimated [9]. The phylogenetic relationships of Asian *Ips* species to other *Ips* species distributed in Europe and America have not been thoroughly investigated [9]. Mitochondrial genome sequencing (mtDNA) is widely used for phylogenetic analyses [14,15,16], but there are few data on mtDNA of bark beetles [17]. In addition, restriction site-associated DNA sequencing based on type IIB restriction enzymes (2b RAD-Seq) is used for cost-effective determination of single nucleotide polymorphisms (SNP) throughout the genome [18,19,20], which has led to promising results in the evolutionary and phylogenetic study of closely related species [21,22,23]. The use of mtDNA and 2b-RAD methods to study the phylogenetic relationships of *Ips* species can be used to clarify their taxonomic status, and also help investigate events related to bark beetle evolution.

Host and mate finding in bark beetles is an integrated process involving pheromones, host and non-host odors, with the aggregation pheromone mediating sex attraction, aggregation behavior, and successful location and infestation of host trees that provide food and oviposition sites [24,25,26]. In each species, these pheromones consist of different blends of chemicals that are usually species specific, and the attractant pheromones of one species may exert a repellent effect on other species, which is closely associated with reproductive isolation [9,27]. The aggregation pheromones of nine Chinese *Ips* species have been identified [28,29,30,31,32,33,34,35,36,37,38], and *Ips* bark beetles mainly use host monoterpenes as biosynthetic precursors of aggregation pheromones [39] or synthesize them de novo via the mevalonate pathway [40,41]. For example, as major components of the aggregation pheromone of *Ips* bark beetles, ipsenol and ipsdienol are biosynthesized de novo [40], while the pheromonal verbenols are derived from host α-pinene [41]. Previous studies have examined pheromone and phylogenetic relationships mainly from the perspective of pheromone composition and quantity [42,43], but we hypothesize that it is more useful to analyze the relationships from the perspective of the biosynthetic origin of pheromones.

In this study, the phylogenetic relationships of *Ips* species collected in China (*Ips typographus* L. 1758, *Ips subelongatus* Motschulsky 1860, *Ips hauseri* Reitter 1984, *Ips nitidus* Eggers 1933, *Ips shangrila* Cognato and Sun 2007, *Ips duplicatus* Sahalberg 1836) were investigated using mtDNA and SNP to explore the evolutionary trend of these Chinese *Ips* species. Then, we constructed phylogenetic trees by combining the mitochondrial cytochrome oxidase I DNA sequence (*COI*) data generated in this study with the data published for *Ips* species in Europe and America to analyze the phylogenetic relationships between the Chinese *Ips* bark beetles and the other *Ips* species. In addition, we analyzed selection pressure, genetic distances, and their correlations with geographic distances of *Ips* species and populations under altitudinal variation using the mitochondrial genome to understand the divergence of evolutionary adaptation. By combining phylogenetic relationships and pheromone differences, we also aimed to analyze the path of pheromone evolution. Finally, we examined differences in mitochondrial structural features and compositions among *Ips* species to determine whether they were related to adaptive genetic evolution.

## 2. Materials and Methods

### 2.1. Specimen Collection and DNA Extraction

We collected live adults of six *Ips* species and one *Dendroctonus* species as a total of 19 populations in China: *Ips typographus*, *Ips subelongatus*, *Ips hauseri*, *Ips nitidus*, *Ips shangrila*, *Ips duplicatus*, and *Dendroctonus micans*, which were determined based on body characteristics (e.g., teeth, body size, frontal tubercles, and number of frontal hairs) and their host plants [44]. Live adults were preserved in anhydrous ethanol and stored in a laboratory freezer at −20 °C until biological analysis. Detailed information on collection sites, hosts, and abbreviated population codes can be found in Table 1. Genomic DNA was extracted from thoracic and leg muscle tissues (five individuals per population) and purified using the Wizard Genomic DNA Purification Kit (Promega Corporation, Madison, WI, USA), according to the manufacturer’s protocol. Then, DNA preparations were quantified using PicoGreen^®^ (Thermo Fisher Scientific, Wilmington, DE, USA) and a NanoDrop 2000 fluorospectrometer (Thermo Fisher Scientific, Wilmington, DE, USA), with DNA integrity detected by 1% agarose gel electrophoresis.

### 2.2. Mitogenome De Novo Sequencing and Assembly

Each genomic sample was sheared using a Covaris M220 ultrasonicator (Covaris, Woburn, MA, USA) to obtain 400–500 bp DNA fragmentation. A 460 bp paired-end library was generated from each sample and sequenced using an Illumina Hiseq X Ten platform (Majorbio Bio-pharm Technology, Shanghai, China) to obtain 4 Gb of data. Reads with low sequencing quality were filtered out using Trimmomatic software (http://www.usadellab.org/cms/?page=trimmomatic (accessed on 1 July 2020)). Raw data were sheared as follows: to delete sequences containing splice fragments and to remove sequences with low mass values (Q < 25). The software Spades [45] was used to assemble the cleaned data and the results were compared by mapping to obtain the longest segments. The mitoZ program [46] was used to annotate the genome of the assembly results. The coding sequence (CDS), transfer RNA (tRNA), and ribosomal RNA (rRNA) regions were filtered out. The mitochondrial genome sequences for each species were submitted to GenBank and the accession numbers are listed in Table 1.

### 2.3. Phylogenetic Analysis

Based on the complete mitochondrial genome sequence, we constructed the maximum likelihood tree (ML) and the maximum parsimony tree (MP) using MEGA v5.2.2 software for the 19 *Ips* populations with *Dendroctonus micans* (Curculionidae, Scolytinae) as an outgroup. The confidence values of each branch node of the phylogenetic tree were tested by bootstrapping with 1000 replicates [47]. We also constructed the Bayesian inference tree (BI) using MrBayes v3.2.2 software (Uppsala, Sweden) [48] and used the optimal alternative model (GTR+G) to build the evolutionary tree. The MCMC method was used to calculate 2,000,000 generations, sampling every 100 generations to ensure sampling independence, and the original 25% of the data were discarded as burn-in. Stationarity was considered achieved when the average standard deviation of split frequencies was less than 0.01 [49]. To illustrate the phylogenetic relationships of *Ips* species within the Scolytinae, we also constructed the ML and BI trees by combining the mitochondrial genomes of 26 published species of Scolytinae (Table S1) and our studied six species with *Curculio elephas* (Curculionidae, Curculioninae) as an outgroup. In addition, a total of 43 *Ips* species with 105 *COI* sequences (19 from our study and 86 from the published literature [9,12,13]) were selected to construct a phylogenetic tree using the BI method with *D. micans, Pseudips concinnus*, and *Pseudips mexicanus* as outgroups (Table S2).

### 2.4. Estimation of Divergence Time

We used the sequences of 13 protein-coding genes to estimate the divergence time for the *Ips* species using the software BEAST v2.3.0 [50]. MrMTgui software was used to select the most appropriate sequence replacement model (GTR) for comparative analyses, and the site heterogeneity model was set to gamma; clock was set to strict clock. The Yule procedure calculated 20,000,000 generations using the MCMC method, sampling every 1000 generations and discarding the first 20% of the data as burn-in. In the absence of relevant fossil records as time markers, the application of the molecular clock was chosen based on the insect mitochondrial genome, which contained the protein-coding genes of *COXI* and had an average substitution rate of 0.00175 substitutions/site/Myr (s/s/Myr) [51]. The substitution rates of other mitochondrial genes were scaled to the mean *COXI* rate, and a weak lognormal distribution with a large standard deviation was used to allow *COXI* calibration to control the analysis. We imported the results generated by the BEAUti 2 software in .xml format into BEAST v2.3.0 to perform the analysis. Then, the Tracer v1.5 software (http://tree.bio.ed.ac.uk/software/tracer (accessed on 1 November 2020)) was used to check the results and obtain a standard distribution based on the effective sample size (ESS) of each estimated parameter, with ESS requiring at least 200 for the posterior and prior. The Tree Annotator V2.3.0 software was used to calculate the consensus tree and annotate the divergence time, and the FigTree V1.3.1 software (http://tree.bio.ed.ac.uk/software/figtree (accessed on 1 December 2020)) was used to open it and check the analysis results.

### 2.5. Comparative Mitogenome Analyses of Bark Beetles

Circle maps of the mitogenome of six *Ips* species (e.g., ItyJG, IsuJM, IshQL, IhaTS, IniXZ, and IduBY) and one *Dendroctonus* species (DmiMX) were generated and displayed using the CGView software (http://stothard.afns.ualberta.ca/downloads/CCT/tutorials.html (accessed on 1 July 2020)). The nucleotide composition, codon usage (except for stop codons), and relative synonymous codon usage (RSCU) of the mitochondrial genomes of the analyzed *Ips* species were calculated using the MEGA 5.2.2 software (Paris, France). The compositional skew was calculated using the following formulas: AT skew = (A − T)/(A + T) and GC skew = (G − C)/(G + C) [52]. To test the interspecific distinctions of the *Ips* species, the numbers of base differences per site between sequences were presented. The analyses included seven nucleotide sequences and the codon positions were 1 + 2 + 3. All positions with less than 95% coverage were eliminated, i.e., less than 5% alignment gaps, missing data, and ambiguous bases were allowed at each position. A total of 14,655 positions were included in the final data set. Evolutionary analyses were performed using the MEGA v5.2.2 software [53].

### 2.6. Genetic Distance, Selection Pressure and Isolation by Distance (IBD) Analysis 

Sequences were preliminarily aligned using the Clustal X program (lllkirch, France) [54]. Pairwise genetic distances were calculated using the MEGA v5.2.2 software based on the Kimura-2 parameter model [55]. The CodeML software implemented in the PAML package was used with the free ratio model to calculate the non-synonymous (Ka) and synonymous (Ks) substitution rates along with each branch of the tree [56]. The selection pressure of the mtDNA genome was assessed by calculating the Ka/Ks ratios of 13 mitochondrially encoded protein genes in different species. The haplotype network of *Ips* species was analyzed using a median-joining algorithm in the program Network 4.6 [57]. We calculated the geographic distance of the sampling sites using the longitude and latitude data and evaluated the correlations between the genetic distance and the geographic distance of the species using the Mantel test of IBD 1.53 [58].

### 2.7. SNP Sequencing and Analysis

In this study, we also sequenced the SNP of the *Ips* populations. The DNA of each sample was extracted using a TIANamp Genomic DNA Kit (Osce Biological Technology, Shanghai, China), and its quality and concentration were determined using the agar-agar assay and a Nanodrop2000 fluorospectrometer. Paired-end sequencing was performed after qualified DNA extraction on the Illumina Hiseq Xten platform (Osce Biological Technology, Shanghai, China). Paired-end sequencing was also performed using 2b-RAD technology, with all samples attached to the enzyme digestion label using the 5′-NNN-3′ linker. Upon completion of sequencing, reads containing restriction sites were extracted from the sequencing data. The Stacks software v 1.34 (Eugene, OR, USA) [59] was used for clustering and reference sequences were generated. The sequencing data were aligned to the reference sequence using the software SOAP v 2.21 (Shenzhen, China) [60]. The methods of haplotype analysis and RAD sequence-based ML tree construction are the same as for mitochondria. The software STRUCTURE v 2.3.4 (Oxford, UK) [61] was used to analyze the population structure from K = 2 to K = 10, and 10 different seeds were selected for 10 repeated analyses. According to the optimal K value determined by the cross-validation error, a box shape plot was drawn for each K value, and the CV value was repeated N times. 

## 3. Results

### 3.1. Phylogenetic Relationships, Divergence Times, and Pheromone Differences in Ips Bark Beetles

**Figure 1:**
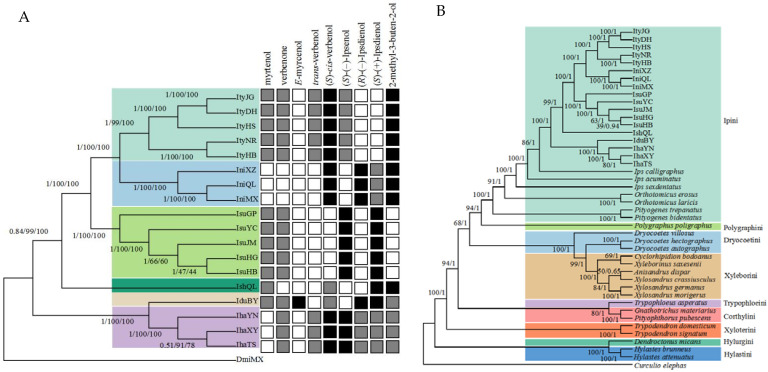
(**A**) Phylogenetic trees (Bayesian tree [BI], maximum parsimony [MP], and maximum likelihood [ML]) of *Ips* bark beetles based on PCGs, with pheromone components superimposed. Numbers above or below branches indicate bootstrap values for BI, ML, and MP, respectively. Different colored clades indicate different species branches. The black squares on the right represent major components of beetle pheromones, the gray squares represent minor components, and the white squares represent beetles that do not use this component as a pheromone. (**B**) Phylogenetic tree (Bayesian tree [BI] and maximum likelihood [ML]) of bark beetles under higher order element based on mtDNA. Numbers above and below branches indicate bootstrap values for BI and ML, respectively.

The phylogenetic trees ML, MP, and BI, which were constructed based on the PCGs of the *Ips* species/populations, showed similar topology with high nodal support in most of the central nodes (Figure 1A). The phylogenetic relationships of the *Ips* species are as follows: (((*I. typographus* + *I. nitidus*) + *I. subelongatus*) + *I. shangrila*) + (*I. duplicatus* + *I. hauseri*). Each species belongs to a separate branch, although *I. typographus* and *I. nitidus* are phylogenetically closely related, and *I. duplicatus* and *I. hauseri* are also closely related. In addition, using *Curculio elephas* as an outgroup, we created ML and BI trees by combining 26 species of Scolytinae (whose mitochondrial genomes were sequenced) with mtDNA sequences of the *Ips* species analyzed in this study (Figure 1B). Initially, the phylogenetic trees based on the mtDNA and the 13 PCGs of the *Ips* species showed a similar topology as in Figure 1A. The phylogenetic relationship within the trees ML and BI was constructed as follows: ((((Ipini + Polygraphini) + (Drycoetini + Xyleborini)) + (Trypophloeini + Corthylini)) + Xyloterini) + (Hylurgini + Hylastini), and the phylogenetic tree exhibited high bootstrap values. The phylogenetic analysis using SNP also showed that the phylogenetic relationships of *Ips* species were similar to those established using the mitochondrial genome (Figure S1).

The results of BEAST dating analysis showed that the age of the youngest common ancestor of *Ips* species was 12.02 Mya, indicating the earliest divergence time of *Ips* species in China. Among them, the divergence time of *I. subelongatus* was estimated to be 6.49 Mya; *I. duplicatus* and *I. hauseri* began to diverge in 4.89 Mya, and *I. typographus* and *I. nitidus* in 1.54 Mya. The *Ips* species have gradually evolved to their present status over the past 400,000 years (Figure S2).

The major and minor pheromone components of *Ips* bark beetles were superimposed on the phylogenetic tree, and a significant difference was found between pheromone components in closely related *Ips* species (Figure 1A). The pheromone components 2-methyl-3-butten-2-ol, (*S*)-(+)-ipsdienol, (*R*)-(–)-ipsdienol, and (*S*)-(–)-ipsenol, which are biosynthesized de novo in the anterior midgut of bark beetles, did not differ gradually among *Ips* species. A similar pattern was observed for the pheromone component (*S*)-*cis*-verbenol, which is derived from a host precursor. It should be noted that the major and minor components of aggregation pheromones of a given species are not tied to a particular synthetic pathway and that interspecific changes in pheromone components do not parallel their evolutionary relationships.

### 3.2. Phylogenetic Relationships among Ips Bark Beetles Worldwide 

**Figure 2:**
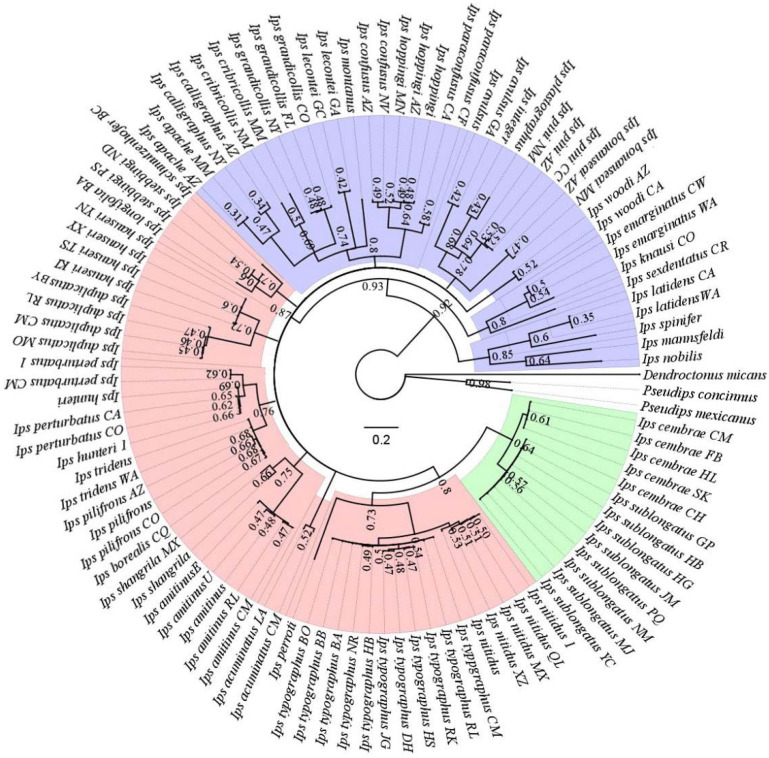
Phylogenetic tree showing 43 *Ips* species based on the *COI* gene. The numbers above the branches indicate the bootstrap values. The different colors in the branches of the phylogenetic tree represent the different hosts of the *Ips* bark beetles: green for larch, pink for spruce, and purple for pine.

**Figure 3:**
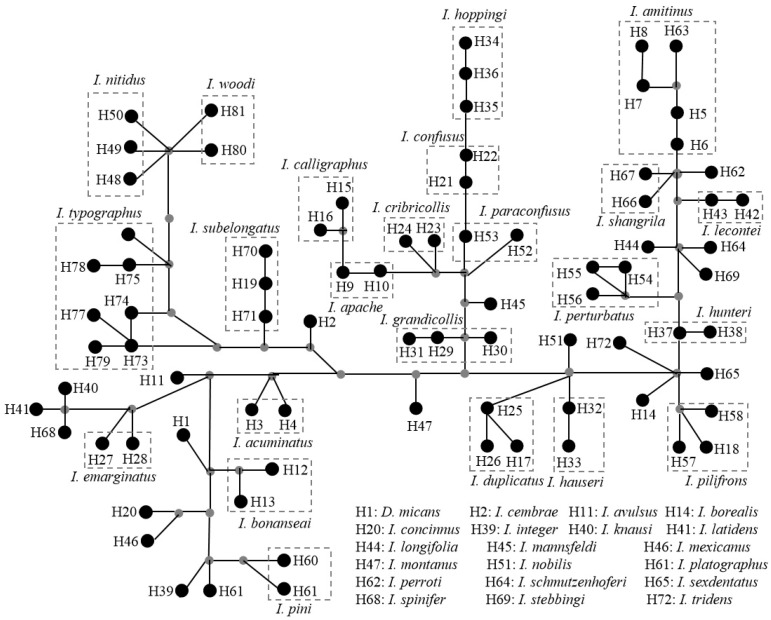
Median-joining network based on the *COI* gene. Each circle represents a haplotype, different areas in the dashed line correspond to different species. The gray dots represent the mutation nodes.

We used the Bayesian method to reconstruct phylogenetic trees based on 105 *COI* sequences for 43 *Ips* species worldwide (Figure 2). The results showed that *Ips* bark beetles were polyphyletic. When we combine the phylogenetic relationships of *Ips* species with the correlations of their hosts, we find that the species are more tightly linked to a specific range and the ranges of the hosts are also more tightly linked. The hosts of spruce, larch, and pine do not overlap, and 23 *Ips* species colonize pine, 18 species feed mainly on spruce, and *I. subelongatus* and *Ips cembrae* Heer 1836 mainly damage larch. We also performed a haplotype analysis based on the 105 *COI* sequences of *Ips* species (Figure 3). A total of 81 haplotypes were identified, including 13 common haplotypes and 48 exclusive haplotypes. The sequences within each common haplotype belong to the same species. The haplotype network results also showed that different haplotypes of the same species were relatively close to each other in the diagram, which intuitively illustrated the relationship between *Ips* species.

### 3.3. Analysis of Selection Pressure, Genetic Distance, and IBD Test

**Figure 4:**
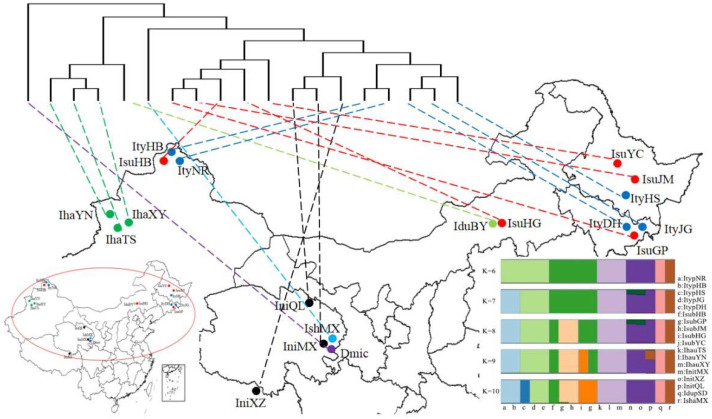
Sampling locations of *Ips* bark beetles. A simplified maximum likelihood tree (ML) based on mtDNA is shown at the top of the figure. The inset in the lower right shows the best genetic clustering (K = 6–10) based on SNP.

**Figure 5:**
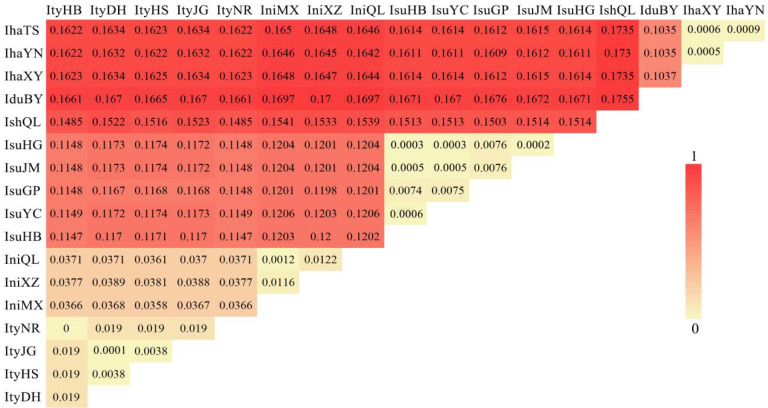
Genetic distance of the six *Ips* species (the deeper the red, the greater the genetic distance).

In this study, the six *Ips* species analyzed are mainly distributed in five mountains in China, which have a wide distribution range and high altitudinal variation (Figure 4). Analysis of selection pressure with 13 PCGs in six *Ips* species revealed a separate Ka/Ks ratio for each terminal branch in the tree ML (Table 2). *Ips nitidus* with the highest altitude distribution had the highest mean (0.0547), but *I. subelongatus* with the lowest altitude distribution retained the lowest mean (0.0186). The same change trends were also observed for *ATP8*, *COII*, *COIII*, *ND1*, *ND3*, *ND4*, *ND4L*, and *ND5* genes. The genetic distance analyses were consistent with the results of the phylogenetic analyses (Figure 5). The genetic distance value between *I. nitidus* and *I. typographus* was the lowest among *Ips* species, indicating their closest genetic relationship; however, the value of *I. shangrila* and *I. duplicatus* was the highest, indicating the most distantly related species. The genetic distances between species were greater than 0.1, except for *I. nitidus* and *I. typographus*, which were greater between species than between populations. The results of the Mantel test showed that there were no significant correlation relationships between genetic distance and geographic distance among species of the genus *Ips*. However, only significant correlations were found between geographic distance and genetic distance between populations of *I. typographus* as well as populations of *I. nitidus* based on different algorithms (Table 3).

### 3.4. Comparative Mitochondrial Genome Analyses of Ips Bark Beetles

**Figure 6:**
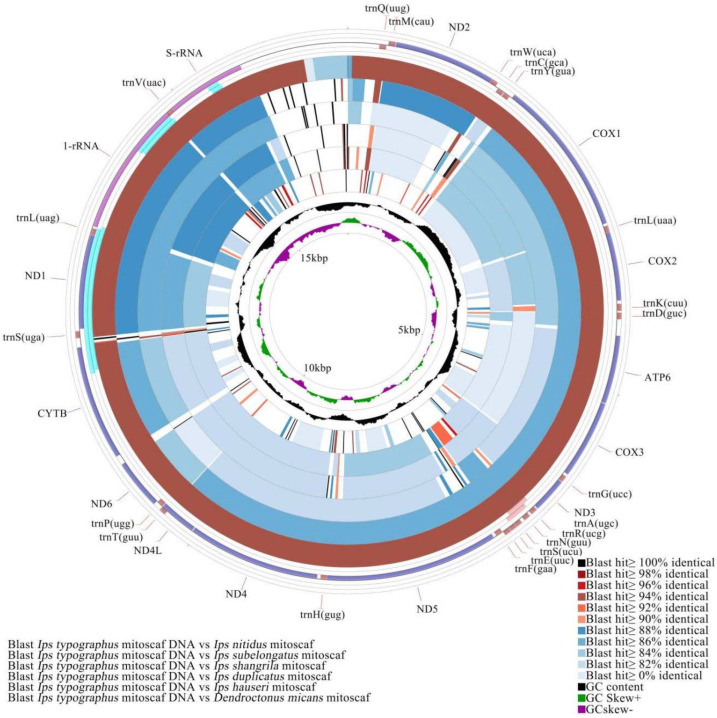
Structure of the mitochondrial genome of bark beetles. The black peak represents GC% divergence and the purple and green peaks represent divergence in GC skew, with green representing positive skew and purple representing negative skew.

We obtained mitogenomic sequences from 19 populations of six *Ips* species with all 36 functional mitochondrial genes except the control region. Sequence lengths vary from 15,259 bp to 15,641 bp and all of these sequences have the same gene arrangement as *Drosophila yakuba* Burla 1954 [62] (Figure S3). We compared the similarity of mitochondrial genome sequences of *Ips* bark beetles, including PCGs, tRNA, and rRNA regions, with those of *I. typographus* (Figure 6). Among them, the sequence similarity of *I. nitidus* was the highest at over 94%; the sequence similarity of *I. hauseri*, *I. duplicatus*, *I. shangrila*, and *I. subelongatus* was mostly between 82% and 88% as compared with that of *I. typographus*; the sequence similarity between *D. micans* and *I. typographus* was practically less than 82%.

Among genes in the entire coding region of the mitochondrial genome of six *Ips* species, the number of spacer regions between adjacent genes ranged from 23 to 27, with the highest number in *I. typographus* and *I. subelongatus*. The number of gene overlap regions ranged from 3 to 5. In the sequence of PCGs, tRNA and rRNA regions of the mitochondrial genome of six *Ips* species, the AT-content was highest in *I. subelongatus*. The values of AT-skew and GC-skew among *Ips* species differed only slightly, and the average AT content in each region of mtDNA was significantly lower than that of *D. micans* (Table S3).

The *ND1* protein-coding genes of *I. subelongatus*, *I. typographus*, *I. hauseri*, and *I. nitidus* all used TTG as the start codon, whereas the others used ATN as the start codon. The *ATP8* gene of six *Ips* species used TAG as the termination codon, whereas most of the other TAA and a few genes used T as the termination codon (Tables S4–S10). We also analyzed the protein-coding genes in the mitochondrial genome, with the amino acids Leu used most frequently and Cys used least frequently. The most frequently used condons were TTT, ATT, and TTA, and AGC with the lowest frequency, which also reflected the AT preference of their nucleotide composition (Figure S4).

## 4. Discussion

### 4.1. Phylogenetic Relationships of Ips Species in Relation to Their Biological Characteristics 

The phylogenetic relationships and evolutionary trend of *Ips* species in China are well established in our study, further confirming the taxonomic status of *I. shangrila* in the genus *Ips* identified by Cognato et al. [63]. We also analyzed the phylogenetic relationship between the six species of *Ips* in China and the other bark beetles in Scolytinae, which was essentially consistent with the reports of Du et al. [17]. The phylogenetic results of *Ips* species are positively correlated with their host ranges. Feng et al. [64] constructed a deep lineage of the conifer genus *Picea*, the host of most *Ips* bark beetles. Here, we compared the phylogenetic relationship between *Ips* bark beetles and their hosts. The main hosts of *I. typographus* and *I. nitudus* are *Picea koraiensis* Nakai 1919 and *Picea crassifolia* Kom. 1923, respectively, which are closely related in the phylogenetic analysis of the hosts. Similarly, the main host of *I. hauseri* is *Picea schrenkiana* Fisch. et Mey., which is genetically distant from Korean spruce and thick-leaved spruce. The host of *I. duplicatus* is *Picea meyeri* Rehd. et Wils. 1914 [32], a species endemic to China, distributed only on the eastern edge of the Hunshandake Sandy Land in Inner Mongolia, and whose systematic status has not yet been determined. The host of *I. subelongatus* is *Larix* sp. (e.g., *Larix gmelinii* Rupr., *Larix kaempferi* Carr., *Larix principis-rupprechtii* Mayr, and *Larix sibirica* Ledeb.), indicating its special taxonomic status within the genus *Ips*. Moreover, the phylogenetic relationship is related to the biological characteristics of *Ips* bark beetles. For example, the body length of *I. hauseri*, *I. duplitatus*, and *I. shangrila* is about 3 mm; in contrast, the body length of *I. typographus* and *I. nitidus* is about 7 mm, and *I. subelongatus* is slightly larger than 7 mm. In addition, the corresponding characteristics in the elevations of the frontal tubercles and the number of frontal hairs (*I. typographus* and *I. nitidus*), and the shape and spacing of the teeth (*I. subelongatus*, *I. hauseri*, *I. duplitatus*, and *I. shangrila*) also help to distinguish the sexes [65,66].

### 4.2. Adaptation of Ips Bark Beetles to a Complex Olfactory Environment

Based on phylogenetic analysis and associated blue stain fungi, *I. subelongatus* (which infests *Larix* sp. in Asia) and *I. cembrae* (which infests *L. decidua* in Europe) were identified as two distinct species [67]. The pheromone components of *I. cembrae* consist of (*S*)-(+)-ipsdienol, (*S*)-(−)-ipsenol, 3-methyl-3-buten-1-ol, and amitinol [68], whereas the pheromones of *I. subelongatus* consist of (*S*)-(+)-ipsdienol and (*S*)-(−)-ipsenol [69,70] and in some regions of China even contain only (*S*)-(−)-ipsenol [30,31], suggesting that pheromones differ in different bark beetle species/populations during evolution. Basically, ipsenol and ipsdienol are partially hydroxylated derivatives of the host myrcene [30,71,72], while the pheromones verbenols (e.g., (*S*)-(−)-*cis*-verbenol, major component of *I. typographus*) are mainly derived from the host α-pinene [1,73]. The large differences in pheromone precursors also suggest an important reason for the differences between species. In addition, *I. nitidus* and *I. shangrila* are sympatric species that share the same host *P. crassifolia* on the Qinghai-Tibet Plateau. The pheromone components of *I. nitidus* (2-methyl-3-buten-2-ol, 74%-(*R*)-(−)-ipsdienol, and (*S*)-(−)-*cis*-verbenol) [33] differed from *I. shangrila* (2-methyl-3-buten-2-ol, 99%-(*S*)-(+)-ipsdienol, and (*S*)-(−)-*cis*-verbenol) [34] in the enantiomeric composition of the critical pheromone component ipsdienol, which was apparently a key factor in maintaining reproductive isolation between these two sympatric species [74]. Based on these pheromone characteristics, we speculate that pheromone blends did not evolve in a typically Darwinian manner through gradual changes in the proportions and structures of the chemicals involved over time, which had been previously assumed [27], but rather it seems more likely that pheromone evolution occurs through a saltatory shift in pheromone components [28,75,76]. Under constant evolutionary pressure (e.g., hosts, climate, altitude, etc.), *Ips* species choose different biological pathways to synthesize their pheromone components to adapt to the changing and complex olfactory environment, directly leading to pheromone divergence and evolution. This adaptive mechanism drives plant–herbivore interactions and also ensures reproductive isolation between sibling species. The results of STURCTURE bloodline analysis have shown that the populations of *I. typographus* and *I. subelongatus* continuously diverge and that the pheromone of *I. subelongatus* exhibits some changes during population differentiation [70,74].

### 4.3. Evolutionary Lineages of the Ips Species in Relation to the Geographic Environment

After analyzing the divergence times, we thought that the evolutionary trend of *Ips* species might be closely related to the geological events in the mountain ranges where they are distributed, as well as to their hosts. The bark beetles of the genus *Ips* evolved in the Miocene when the Qinghai-Tibet Plateau and the Tienshan Mountains were uplifted. Geological events caused their ancestors to seek refuge and gradually split into new species as they adapted to the new habitat. The evolution and dispersal of their hosts was also influenced by geological events at an appropriate time [77]. The uplift of the Qinghai-Tibet Plateau and the Tienshan Mountains from the Miocene to the Pliocene accelerated the climatic drought and Asian monsoon within the Asian continent [78], which played an important role in the spread of spruce/larch [79]. Similar results were found for other highland species in China, such as the Asian honeybee [80] and locust [81].

Since most bark beetle species in China are distributed in a wide range of elevations, some of them at high or even very high elevations, the genetic adaptation mechanisms of *Ips* bark beetles to high elevation environments were also analyzed from the perspective of mitogenomics. We found that selection pressure is significantly higher in *I. nitidus* and *I. shangrila*, which are common at higher elevations, than in *I. typographus* and *I.* *subelongatus*, which are common at lower elevations. This suggests that the low oxygen and cold environment at higher elevations causes selection on the mitochondrial gene of *Ips* species. More than 95% of the total cellular energy in eukaryotic cells is generated by mitochondrial OXPHOS [82]. Proteins encoded by 13 mitochondrial genes play a key role in adaptation to extreme, high-altitude environments [83,84]. The results of selection pressure reflect the adaptability of mitochondrial genes of *Ips* species to different microhabitats. Similar results have been found in high-altitude species such as Tibetan wild yak [85], Tibetan human [86], and schizothoracine fish [87].

The results of genetic distance of *Ips* species were consistent with those of phylogenetic trees, with significantly less genetic distance within species than between species. There were no significant correlations between geographic distance and genetic distance among *Ips* species, but they are closely related to their ranges, hosts, and biological characteristics. *Ips typographus* and *I. subelongatus* have stronger flight ability and a wider host range than other *Ips* species, resulting in a relatively small genetic distance between them and other species; in contrast, *Ips duplicatus*, *I. hauseri*, and *I. shangrila* have poorer flight ability and breed only in species of the genus *Picea*. The barriers between the Tarim Basin, the Qilian Mountains, and the Plateau increase the genetic distance between them and other species. In contrast, the genetic distance between *I. nitidus* and *I. typographus* was small, suggesting that this is the result of the evolution of *I. nitidus* to adapt to the extreme environment. The fact that the divergence time of *I. nitidus* and *I. typographus* not sympatric species, is the youngest in our analyses confirms their relatedness. 

### 4.4. Adaptive Evolution Inferred from Comparative Mitochondrial Genome ANALYSIS

The base content of the entire mitochondrial genome of six *Ips* species follows the same pattern: A > T > C > G, with the AT content being significantly higher than the GC content, similar to the species *I. grandicllis* and *I. sexdentatus* [88]. The sorting order of gene sequences and the use of start and end codons are also consistent with the reported species (*Ips grandicollis*, *Ips typographus*, and *Ips sexdentatus*) [88], suggesting evolutionary conservatism of the mitochondrial genome in *Ips* species [82]. The results of sequence consistency comparisons among *Ips* species are identical to those of phylogenetic trees. For example, the sequence consistency of *I. nitidus* and *I. typographus* is over 94% in each region of the mitochondrial genome, the similarity between *I. duplitatus* and *I. hauseri* is relatively high, and *I. shangrila* and *I. subelongatus* also regularly agree with phylogenetic relationships. In addition, the frequency of amino acid codon use in *I. subelongatus* appears to differ from that of other species, suggesting that it may be related to its larch host.

### 4.5. Phylogenetic Status of Ips Species from China Based on COI Gene Analysis

In studying the phylogenetic and taxonomic relationships between Chinese and European and American *Ips* species based on *COI* sequences, we found that the species groups in the current classification for *Ips* are not monophyletic. The Chinese *Ips* species do not form a monophyletic group, which are distributed among the European and North American species in the phylogenetic tree. The same conclusions were drawn for the European *Ips* bark beetles [9]. Therefore, the phylogenetic relationships of *Ips* species on the same continent are not more distant from each other than those on other continents, i.e., geographic distance is not a determining factor for phylogenetic relationships. From the early Miocene to the Pliocene, East Asia and western North America were connected by the Bering land bridge, which was covered with temperate conifer forests [89]. Conifer-related insect groups such as the bark beetles *Ips* may have established widespread populations on these continents at this time. Multiple invasions between American and Eurasian *Ips* species, as well as the fact that most *Ips* species also interbred, could account for polyphyletic groups [90]. The phylogenetic relationships of *Ips* species are related to their hosts, with sibling host species being phylogenetically closely related. Of course, different *Ips* species may share the same host, which may be related to adaptive evolution between *Ips* beetles and hosts. *Ips borealis* Swaine 1911, *I. hunteri* Swaine 1917, *I. pilifrons* Swaine 1912, and *I. tridens* Mannerheim 1852 are morphologically very similar and difficult to distinguish, and our results suggest that these beetles are closely related and can be resolved by phylogenetic analyses. We hypothesize that *I. knausi* Swaine 1915, *I. emarginatus* LeConte 1876, and *I. sexdentatus* Boerner 1767 are basal species of the genus *Ips*. To date, mitochondrial genome sequences of *Ips* species are relatively sparse and incomplete, limiting phylogenetic analysis to distinguish monophyletic relationships and determine phylogenetic relationships. Stauffer et al. [13] hypothesized that speciation of *I. acuminatus* was accompanied by host switching from *Picea abies* to *Pinus* *silvestris* L.; however, we hypothesize that *Pinus* trees are the original hosts of bark beetles and that they were also the hosts of the basal species *Ips latidens*, *Ips spinifer*, *Ips mannsfeldi*, and *Ips nobilis*, which is consistent with the conclusion of Cognato et al. [9]. The genus *Ips* split from the genus *Dendroctonus* 60 Mya ago [91], and the genus *Picea* gradually separated more recently, resulting in different *Ips* species adapting to host switching and gradually forming the present status quo in the process of host dispersal and evolution. The host switch of *Dendroctonus* spp. from *Pinus*-associated ancestors to *Larix* and *Picea* is thought to have occurred simultaneously in the western Nearctic regions after the late Oligocene/early Miocene [92], which is also consistent with the timing of the *Ips* divergence. The ancestors of some *Ips* species did not breed in species of the genus *Picea* [9], as *Ips latidens*, *Ips spinifer*, *Ips mannsfeldi*, and *Ips nobilis* are basal to *Ips* and all breed in the *Pinus* species. Further studies are needed to refine estimates of the age of these events. Independent studies of different insect species associated with conifers tend to have relatively similar biogeographic and host evolutionary scenarios, which adds to their credibility [93].

## 5. Conclusions

In our study, we sequenced the mitogenomes of *Ips* bark beetles from 19 populations, enriched the baseline data, and examined their phylogenetic and evolutionary relationships. *Ips* species show adaptive evolution under the influence of the uplift of the Qinghai-Tibet Plateau and the Tienshan Mountains, consistent with the evolution of their hosts, as indicated by interspecific divergence time, genetic distance, selection pressure, and comparison of mitochondrial genomes. The phylogenetic relationship between species and the evolutionary relationship of pheromones are also discussed from a new perspective, namely the source of pheromone synthesis, which provides a new idea to study their relationships. Our results provide a better understanding of the mechanisms of adaptive evolution in relation to the environment, hosts, and phenotypes of bark beetles.

## Figures and Tables

**Table 1:** The information list of specimens collected in China.

Species	Code	Location (District, Province)	Latitude	Longitude	Altitude	Hosts	Accession Number
*Ips typographus*	ItyJG	Yanbian, Jilin	43.35	130.18	660	*Picea koraiensis* Nakai	OK110249
	ItyHB	Habahe, Xinjiang	48.47	86.68	994	*P. obovata* Ledeb.	OK147100
	ItyHS	Hengshan, Jilin	46.24	128.95	175	*P. koraiensis* Nakai	OK147101
	ItyDH	Dunhua, Jilin	43.21	127.65	575	*P. koraiensis* Nakai	OK147111
	ItyNR	Naren, Xinjiang	48.47	86.69	1024	*P. obovata* Ledeb.	OK147109
*Ips subelongatus*	IsuHB	Habahe, Xinjiang	48.39	86.79	1329	*Larix sibirica* Ledeb.	OK110250
	IsuGP	Helong, Jilin	42.04	128.8	572	*L. olgensis* Henry	OK086802
	IsuJM	Jiamusi, Heilongjiang	46.43	130.65	192	*L. olgensis* Henry	OK147102
	IsuHG	Chifeng, Inner Mongolia	43.57	117.52	1447	*L. principis-rupprechtii* Mayr	OK147107
	IsuYC	Yichun, Heilongjiang	48.65	126.63	273	*L. gmelinii* Rupr.	OK147112
*Ips hauseri*	IhaTS	Tienshan, Xinjiang	43.18	82.85	1516	*P. schrenkiana* Fisch. et Mey.	OK147104
	IhaYN	Yining, Xinjiang	44.2	81.67	2016	*P. schrenkiana* Fisch. et Mey.	OK147105
	IhaXY	Xinyuan, Xinjiang	43.27	84.3	1654	*P. schrenkiana* Fisch. et Mey.	OK147106
*Ips nitidus*	IniMX	Maixiu, Qinghai	35.26	101.89	3156	*P. crassifolia* Kom.	OK147110
	IniXZ	Leiwuqi, Xizang	31.25	96.49	3947	*P. likiangensis* Pritz.	OK147108
	IniQL	Qilian, Qinghai	38.16	100.49	3407	*P. crassifolia* Kom.	OK147114
*Ips shangrila*	IshQL	Qilian, Qinghai	35.26	101.89	3156	*P. crassifolia* Kom.	OK147103
*Ips duplicatus*	IduBY	Chifeng, Inner Mongolia	43.53	117.22	1357	*P. meyeri* Rehd. et Wils.	OK147102
*Dendroctonus micans*	DmiMX	Maixiu, Qinghai	35.27	101.91	3050	*P. purpurea* Mast.	OL147113

**Table 2:** Ka/Ks values for each mitochondrial protein-coding gene of six species.

Species	*ATP6*	*ATP8*	*COI*	*COII*	*COIII*	*Cytb*	*ND1*	*ND2*	*ND3*	*ND4*	*ND4L*	*ND5*	*ND6*	Average
*I. nitidus*	0.0304	0.0219	0.0302	0.0473	0.0356	0.0603	0.0651	0.0693	0.0298	0.0220	0.0096	0.0621	0.2278	0.0547
*I. shangrila*	0.0334	0.0209	0.0159	0.0387	0.0894	0.0298	0.0414	0.0719	0.0530	0.0202	0.0203	0.0604	0.0353	0.0408
*I. duplicatus*	0.0315	0.0245	0.0050	0.0636	0.0322	0.0310	0.0507	0.1258	0.0461	0.0326	0.0540	0.0757	0.0485	0.0478
*I. hauseri*	0.0220	0.0196	0.0129	0.0128	0.0111	0.0118	0.0531	0.0566	0.0259	0.0339	0.0116	0.0521	0.0633	0.0297
*I. typgraphus*	0.0272	0.0001	0.0122	0.0001	0.0001	0.0240	0.0261	0.0897	0.0001	0.0001	0.0001	0.0216	0.1252	0.0251
*I. subelongatus*	0.0101	0.0001	0.0001	0.0001	0.0163	0.0287	0.0150	0.0381	0.0060	0.0001	0.0012	0.0362	0.0899	0.0186

**Table 3:** The results of the Mantel test between genetic distance and geographic distance.

	Formula Mode	*r* (Correlation Coefficient)	*p*-Value
*Ips* (between all species)	genetic distance & geographic distance	0.0526	0.198
	genetic distance & log(geographic distance)	0.105	0.097
	log(genetic distance) & geographic distance	0.1383	0.07
	log(genetic distance) & log(geographic distance)	0.2282	0.017
*I. typographus*	genetic distance & geographic distance	0.9943	0.019
	genetic distance & log(geographic distance)	0.8111	0.019
	log(genetic distance) & geographic distance	0.8441	0.047
	log(genetic distance) & log(geographic distance)	0.8402	0.001
*I. subelongatus*	genetic distance & geographic distance	−0.1942	0.357
	genetic distance & log(geographic distance)	−0.2108	0.559
	log(genetic distance) & geographic distance	−0.1508	0.384
	log(genetic distance) & log(geographic distance)	−0.1938	0.487
*I. nitidus*	genetic distance & geographic distance	0.9528	0.015
	genetic distance & log(geographic distance)	0.9786	0.001
	log(genetic distance) & geographic distance	0.9432	0.001
	log(genetic distance) & log(geographic distance)	0.9720	0.001
*I. hauseri*	genetic distance & geographic distance	−0.0857	0.508
	genetic distance & log(geographic distance)	−0.0068	0.346
	log(genetic distance) & geographic distance	−0.0414	0.508
	log(genetic distance) & log(geographic distance)	0.0376	0.508

## Data Availability

All data in this article are deposited in GenBank under the accession numbers listed in Table 1.

## Supplementary Materials

The following supplementary information can be downloaded from https://www.mdpi.com/article/10.3390/biology11030384/s1. Figure S1: Phylogenetic tree (ML) of *Ips* geographic populations based on SNP. Numbers above or below branches indicate bootstrap values, Figure S2: Tree of divergence times of *Ips* species/populations. The numbers above the branches indicate the divergence time (in millions of years) of the branches. The middle solid line indicates the mean population size, and the gray area below and above the solid line indicates the 95% confidence interval, Figure S3: Circular maps of six newly sequenced bark beetles. Genes that were transcribed clockwise are shown outside the circle, while genes that were transcribed counterclockwise are shown inside the circle, Figure S4: Amino acid usage of protein-coding genes of the mitochondrial genome in the genus *Ips*, Table S1: The list of sample information of mitochondrial genomes of 26 published species of Scolytidae, Table S2: *Ips* species used in this study, Table S3: Base composition in mitochondrial genomes of bark beetles, Table S4: Organization of the mitochondrial genome of *I. typographus*, Table S5: Organization of the mitochondrial genome of *I. subelongatus*, Table S6: Organization of the mitochondrial genome of *I. shangrila*, Table S7: Organization of the mitochondrial genome of *I. duplicatus*, Table S8: Organization of the mitochondrial genome of *I. hauseri*, Table S9: Organization of the mitochondrial genome of *I. nitidus*, Table S10: Organization of the mitochondrial genome of *D. micans*.

## References

[1] Fang J.X., Liu M., Zhang S.F., Liu F., Zhang Z., Zhang Q.H., Kong X.B. (2020). Chemical signal interactions of the bark beetle with fungal symbionts, and host/non-host trees. J. Exp. Bot..

[2] Byers J.A. (1983). Bark beetle conversion of a plant compound to a sex-specific inhibitor of pheromone attraction. Science.

[3] Fang J.X., Zhang S.F., Liu F., Zhang Z., Cheng B., Zhang Q.H., Kong X.B. (2021). Functional investigation of monoterpenes for improved understanding of the relationship between hosts and bark beetles. J. Appl. Entomol..

[4] Rosenberger D.W., Venette R.C., Aukema B.H. (2018). Development of an aggressive bark beetle on novel hosts: Implications for outbreaks in an invaded range. J. Appl. Ecol..

[5] Therrien J., Mason C.J., Cale J.A., Adams A., Aukema B.H., Currie C.R., Raffa K.F., Erbilgin N. (2015). Bacteria influence mountain pine beetle brood development through interactions with symbiotic and antagonistic fungi: Implications for climate-driven host range expansion. Oecologia.

[6] Bracewell R.R., Vanderpool D., Good J.M., Six D.L. (2018). Cascading speciation among mutualists and antagonists in a tree–beetle–fungi interaction. Proc. R. Soc. B-Biol. Sci..

[7] Raffa K.F., Aukema B.H., Bentz B.J., Carroll A.L., Hicke J.A., Turner M.G., Romme W.H. (2008). Cross-scale drivers of natural disturbances prone to anthropogenic amplification: The dynamics of bark beetle eruptions. BioScience.

[8] Biedermann P.H.W., Müller J., Grégoire J.C., Gruppe A., Hagge J., Hammerbacher A., Hofstetter R.W., Kandasamy D., Kolarik M., Kostovcik M. (2019). Bark beetle population dynamics in the Anthropocene: Challenges and solution. Trends Ecol. Evol..

[9] Cognato A.I., Felix A. (2000). Phylogeny of *Ips* DeGeer species (Coleoptera: Scolytidae) inferred from Mitochondrial Cytochrome Oxidase I DNA sequence. Mol. Phylogenet. Evol..

[10] Cognato A.I., Seybold S.J., Sperling F.A.H. (1999). Incomplete barriers to mitochondrial gene flow between pheromone races of the North American pine engraver, *Ips pini* (Say) (Coleoptera: Scolytidae). Proc. R. Soc. B-Biol. Sci..

[11] Cognato A.I. (2000). Phylogenetic analysis reveals new genus of Ipini bark beetle (Scolytidae). Ann. Entomol. Soc. Am..

[12] Bertheau C., Schuler H., Arthofer W., Avtzis D.N., Mayer F., Krumböck S., Moodley Y., Stauffer C. (2013). Divergent evolutionary histories of two sympatric spruce bark beetle species. Mol. Ecol..

[13] Stauffer C., Lakatos F., Hewitt G.M. (1997). The phylogenetic relationships of seven European *Ips* (Scolytidae, Ipinae) species. Insect Mol. Biol..

[14] Lin C.P., Danforth B.N. (2004). How do insect nuclear and mitochondrial gene substitution patterns differ? Insights from Bayesian analyses of combined datasets. Mol. Phylogenet. Evol..

[15] Gissi C., Iannelli F., Pesole G. (2008). Evolution of the mitochondrial genome of Metazoa as exemplified by comparison of congeneric species. Heredity.

[16] Mayer F., Piel F.B., Cassel-Lundhagen A., Kirichenko N., Grumiau L., Økland B., Bertheau C., Grégoire J.C., Mardulyn P. (2015). Comparative multi-locus phylogeography of two Palaearctic spruce bark beetles: Influence of contrasting ecological strategies on genetic variation. Mol. Ecol..

[17] Du H.C., Fang J.X., Shi X., Zhang S.F., Liu F., Yu C.M., Zhang Z., Kong X.B. (2021). Comparative analysis of eight mitogenomes of bark beetles and their phylogenetic implicaitons. Insects.

[18] Wang S., Meyer E., McKay J.K., Matz M.V. (2012). 2b-RAD: A simple and flexible method for genome-wide genotyping. Nat. Methods.

[19] Seetharam A.S., Stuart G.W. (2013). Whole genome phylogeny for 21 *Drosophila* species using predicted 2b-RAD fragments. PeerJ.

[20] Du Z.Y., Hasegawa H., Cooley J.R., Simon C., Yoshimura J., Cai W.Z., Li H. (2019). Mitochondrial genomics reveals shared phylogeographic patterns and demographic history among three periodical cicada species groups. Mol. Biol. Evol..

[21] Puritz J.B., Matz M.V., Toonen R.J., Weber J.N., Bolnick D.I., Bird C.E. (2014). Demystifying the RAD fad. Mol. Ecol..

[22] Arbizu C.I., Ellison S.L., Senalik D., Simon P.W., Spooner D.M. (2016). Genotyping-by-sequencing provides the discriminating power to investigate the subspecies of *Daucus carota* (Apiaceae). BMC Evol. Biol..

[23] Diaz-Arce N., Arrizabalaga H., Murua H., Irigoien X., Rodriguez E.N. (2016). RAD-seq derived genome-wide nuclear markers resolve the phylogeny of tunas. Mol. Phylogenet. Evol..

[24] Kirkendall L.R., Biedermann P.H.W., Jordal B.H., Vega F.E., Hofstetter R.W. (2015). Evolution and Diversity of Bark and Ambrosia Beetles. Bark Beetles: Biology and Ecology of Native and Invasive Species.

[25] Hulcr J., Atkin Son T.H., Cognato A.I., Jordal B.H., McKenna D.D., Vega F.E., Hofstetter R.W. (2015). Morphology, taxonomy an phylogenetics of bark beetles. Bark Beetles: Biology and Ecology of Native and Invasive Species.

[26] Hou X.Q., Yuvaraj J.K., Roberts R.E., Zhang D.D., Unelius C.R., Lofstedt C., Andersson M.N. (2021). Functional evolution of a bark beetle odorant receptor clade detecting monoterpenoids of different ecological origins. Mol. Biol. Evol..

[27] Roelofs W.L., Brown R.L. (1982). Pheromones and evolutionary relationships of Tortricidae. Annu. Rev. Ecol. Syst..

[28] Symonds M.R.E., Elgar M.A. (2004). The mode of pheromone evolution: Evidence from bark beetle. Proc. R. Soc. B-Biol. Sci..

[29] Guo H., Lackus N., Köllner T.G., Li R., Bing J., Wang Y., Baldwin I.T., Xu S. (2020). Evolution of a novel and adaptive floral scent in wild tobacco. Mol. Biol. Evol..

[30] Zhang Q.H., Birgersson G., Schlyter F., Chen G.F. (2000). Pheromone components in the larch bark beetle, *Ips cembrae*, from China: Quantitative variation among attack phases and individuals. J. Chem. Ecol..

[31] Zhang Q.H., Schlyter F., Chen G., Wang Y. (2007). Electrophysiological and behavioral responses of *Ips subelongatus* to semiochemicals from its hosts, non-hosts, and conspecifics in China. J. Chem. Ecol..

[32] Zhang Q.H., Schlyter F., Liu G.T., Sheng M.L., Birgersson G. (2007). Electrophysiological and behavioral responses of *Ips duplicatus* to aggregation pheromone in Inner Mongolia, China: Amitinol as a potential pheromone component. J. Chem. Ecol..

[33] Zhang Q.H., Ma J.H., Zhao F.Y., Song L.W., Sun J.H. (2009). Aggregation pheromone of the Qinghai spruce bark beetle, *Ips nitidus* eggers. J. Chem. Ecol..

[34] Zhang Q.H., Song L.W., Ma J.H., Han F.Z., Sun J.H. (2009). Aggregation pheromone of a newly described spruce bark beetle, *Ips shangrila* Cognato and Sun from China. Chemoecology.

[35] Bakke A. (1976). Spruce bark beetle, *Ips typographus*: Pheromone production and field response to synthetic pheromones. Naturwissenschafren.

[36] Fang J.X., Du H.C., Shi X., Zhang S.F., Liu F., Zhang Z., Zu P.J., Kong X.B. (2021). Monoterpenoid signals and their transcriptional responses to feeding and juvenile hormone regulation in bark beetle *Ips huaseri*. J. Exp. Biol..

[37] Eidmann H.H., Birgersson G. (1988). Semiochemicals in the East Himalaya spruce bark beetle. Anz. Schädlingskunde Pflanzenschutz Umweltschutz.

[38] Vité J., Bakke A., Renwick J.A.A. (1972). Pheromones in *Ips* (Coleoptera: Scolytidae): Occurrence and production. Can. Entomol..

[39] Tittiger C., Blomquist G.J. (2017). Pheromone biosynthesis in bark beetles. Curr. Opin. Insect Sci..

[40] Ivarsson P., Schlyter F., Birgersson G. (1993). Demonstration of *de novo* pheromone biosynthesis in *Ips duplicatus* (Coleoptera: Scolytidae): Inhibition of ipsdienol and *E*-myrcenol production by compaction. Insect Biochem. Mol. Biol..

[41] Seybold S.J., Quilici D.R., Tillman J.A., Vanderwel D., Wood D.L., Blomquist G.J. (1995). *De novo* biosynthesis of the aggregation pheromone components ipsenol and ipsdienol by the pine bark beetles *Ips paraconfusus* Lanier and *Ips pini* (Say) (Coleoptera:Scolytidae). Proc. Natl. Acad. Sci. USA.

[42] Cane J.H., Stock M.W., Wood D.L., Gast S.J. (1990). Phylogenetic relationship of *Ips* bark beetles (Coleoptera: Scolytidae): Electrophoretic and morphometric analyses of the grandicollis group. Biochem. Syst. Ecol..

[43] Cognato A.I., Seybold S.J., Wood D.L., Teale S.A. (1997). A cladistic analysis of pheromone evolution in *Ips* bark beetles (Coleoptera: Scolytidae). Evolution.

[44] Huang F.S., Lu J. (2015). The Classification Outline of Scolytidae from China.

[45] Dmitry A., Anton K., Jeffrey S., McLean P.A., Pevzner A.N. (2016). HYBRIDSPADES: An algorithm for hybrid assembly of short and long reads. Bioinformatics.

[46] Meng G., Li Y., Yang C., Liu S. (2019). MitoZ: A toolkit for animal mitochondrial genome assembly, annotation and visualization. Nucleic Acid Res..

[47] Guindon S., Gascuel O. (2003). A simple, fast, and accurate algorithm to estimate large phylogenies by maximum likelihood. Syst. Biol..

[48] Ronquist F., Huelsenbeck J.P. (2003). MrBayes 3: Bayesian phylogenetic inference under mixed models. Bioinformatics.

[49] Huelsenbeck J.P., Ronquist F., Nielsen R., Bollback J.P. (2011). Bayesian inference of phylogeny and its impact on evolutionary biology. Science.

[50] Bouckaert R., Heled J., Kühnert D., Vaughan T., Wu C.H., Xie D., Suchard M., Rambaut A., Drummond A.J. (2014). BEAST 2: A software platform for bayesian evolutionary analysis. PLoS Comput. Biol..

[51] Papadopoulou A., Anastasiou I., Vogler A.P. (2010). Revisiting the insect mitochondrial molecular clock: The mid-Aegean trench Calibration. Mol. Biol. Evol..

[52] Perna N.T., Kocher T.D. (1995). Patterns of nucleotide composition at fourfold degenerate sites of animal mitochondrial genomes. J. Mol. Evol..

[53] Tamura K. (2011). MEGA5: Molecular evolutionary genetics analysis using maximum likelihood, evolutionary distance, and maximum parsimony methods. Mol. Biol. Evol..

[54] Thompson J.D., Gibson T.J., Plewniak F., Jeanmougin F., Higgins D.G. (1997). The CLUSTAL X Windows interface: Flexible strategies for multiple sequence alignment aided by quality analysis tools. Nucleic Acids Res..

[55] Kimura M. (1980). A simple method for estimating evolutionary rates of base substitutions through comparative studies of nucleotide sequences. J. Mol. Evol..

[56] Yang Z. (2007). PAML 4: Phylogenetic analysis by maximum likelihood. Mol. Biol. Evol..

[57] Bandelt H., Forster P., Röhl A. (1999). Median-joining networks for inferring intraspecifific phylogenies. Mol. Biol. Evol..

[58] Bohonak A.J. (2002). IBD (Isolation by Distance): A program for analyses of isolation by distance. J. Hered..

[59] Catchen J., Hohenlohe P.A., Bassham S. (2013). Stacks: An analysis tool set for population genomics. Mol. Ecol..

[60] Li R., Yu C., Li Y., Lam T.W., Yiu S.M., Kristiansen K., Wang J. (2009). SOAP2: An improved ultrafast tool for short read alignment. Bioinformatics.

[61] Pritchard J.K., Stephens M.J., Donnelly P.J. (2000). Inference of population structure using multilocus genotype data. Genetics.

[62] Clary D.O., Wolstenholme D.R. (1985). The ribosomal RNA genes of *Drosophila* mitochondrial DNA. Nucleic Acids Res..

[63] Cognato A.I., Sun J.H. (2007). DNA based cladograms augment the discovery of a new *Ips* species from China (Coleoptera: Curculionidae: Scolytinae). Cladistics.

[64] Feng S., Ru D., Sun Y., Mao K.S., Milne R., Liu J.Q. (2019). Trans-lineage polymorphism and nonbifrucating diversification of the genus *Picea*. New Phytol..

[65] Shi X., Shen J.C., Zhang S.F., Liu F., Xu F.Y., Wang G.L., Zhang Z., Kong X.B. (2020). Comparative analysis of the type and number of larval sensilla on the antennae and mouthparts of *Ips typographus* and *Ips subelongatus* using SEM. Zool. Anz..

[66] Shi X., Zhang S.F., Liu F., Xu F.Y., Zhang F.B., Guo X.B., Zhang Z., Kong X.B. (2020). SEM analysis of sensilla on the mouthparts and antennae of Asian larch bark beetle *Ips subelongatus*. Micron.

[67] Stauffer C., Kirisits T., Nussbaumer C., Pavlin R., Wingfield M.J. (2001). Phylogenetic relationships between the European and Asian eight spined larch bark beetle populations (Coleoptera, Scolytidae) inferred from DNA sequences and fungal associates. Eur. J. Entomol..

[68] Kohnle U., Vité J., Erbacher C., Bartels J., Francke W. (1988). Aggregation response of European engraver beetles of the genus *Ips* mediated by terpenoid pheromones. Entomol. Exp. Appl..

[69] Song L.W., Zhang Q.H., Chen Y.Q., Zuo T.T., Ren B.Z. (2011). Field responses of the Asian larch bark beetle, *Ips subelongatus*, to potential aggregation pheromone components: Disparity between two populations in northeastern China. Insect Sci..

[70] Chen D.F., Li Y.J., Zhang Q.H., Zhang S.F., Wang H.B., Zhang Z., Zhao L.L., Kong X.B. (2016). Population divergence of aggregation pheromone responses in *Ips subelongatus* in Northeastern China. Insect Sci..

[71] Byers J.A., Birgersson G. (1990). Pheromone production in a bark beetle independent of myrcene precursor in host pine species. Naturwissenschaften.

[72] Schlyter F., Birgersson G., Byers J.A., Bakke A. (1992). The aggregation pheromone of *Ips duplicatus* and its role in competitive interactions with *I. typographus*. Chemoecology.

[73] Renwick J., Hughes P., Krull I. (1976). Selective production of *cis*- and *trans*-verbenol from (–)-and (+)-alpha by a bark beetle. Science.

[74] Schlyter F., Jakus R., Han F.Z., Ma J.H., Kalinová B., Mezei P., Sun J.H., Ujhelyiová L., Zhang Q.H. (2015). Reproductive Isolation of *Ips nitidus* and *I. shangrila* in Mountain Forests of Western China: Responses to Chiral and Achiral Candidate Pheromone Components. J. Chem. Ecol..

[75] Roelofs W.L., Liu W.T., Hao G.X., Jiao H.M., Rooney A.P., Linn C.E. (2002). Evolution of moth sex pheromones via ancestral genes. Proc. Natl. Acad. Sci. USA.

[76] Baker T.C. (2002). Mechanism for saltational shifts in pheromone communication systems. Proc. Natl. Acad. Sci. USA.

[77] Sun Y., Abbott R., Li L., Zou J., Liu J. (2014). Evolutionary history of purple cone spruce (*Picea purpurea*) in the Qinghai-Tibet Plateau: Homoploidhybrid origin and pleistocene expansion. Mol. Ecol..

[78] An Z.S., Kutzbach J.E., Prell W.L., Porter S.C. (2001). Evolution of Asian monsoons an phased uplift of the Himalaya Tibetan Plateau since late miocene times. Nature.

[79] Li L.L., Sun Y.S., Zou J.B., Wei Y., Wang X., Liu J.Q. (2015). Origin and speciation of *Picea schrenkiana* and *Picea smithiana* in the center Asian highlands and himalayas. Plant Mol. Biol. Rep..

[80] Santiago M., Ken T., Matthew J.C., Anna O., Carles V., Andreas W., Matthew T.W. (2019). The genomic basis of adaptation to high-altitude habitats in the eastern honey bee (Apis cerana). Mol. Ecol..

[81] Sun J.T., Duan X.Z., Hoffmann A.A., Liu Y., Garvin M.R., Chen L., Hu G., Zhou J.C., Huang H.J., Xue X.F. (2019). Mitochondrial variation in small brown planthoppers linked to multiple traits and probably reflecting a complex evolutionary trajectory. Mol. Ecol..

[82] Boore J.L. (1999). Animal mitochondrial genomes. Nucleic Acids Res..

[83] Shen Y.Y., Liang L., Zhu Z.H., Zhou W.P., Irwin D., Zhang Y.P. (2010). Adaptive evolution of energy metabolism genes and the origin of flight in bats. Proc. Natl. Acad. Sci. USA.

[84] Sun Y.B., Shen Y.Y., Irwin D.M., Zhang Y.P. (2011). Evaluating the roles of energetic functional constraints on teleost mitochondrial encoded protein evolution. Mol. Biol. Evol..

[85] Wang Z., Yonezawa T., Liu B., Ma T., Shen X., Su J.P., Guo S.C., Hasegawa M., Liu J.Q. (2011). Domestication relaxed selective constraints on the yak mitochondrial genome. Mol. Biol. Evol..

[86] Gu M.L., Dong X.Q., Shi L.I., Shi L., Lin K., Huang X.Q., Chu J.Y. (2012). Differences in mtDNA whole sequence between Tibetan and Han populations suggesting adaptive selection to high altitude. Gene.

[87] Li Y., Ren Z.M., Shedlock A.M., Wu J.Q., Sang L., Tersing T., Hasegawa M., Yonezawa T., Zhong Y. (2013). High altitude adaptation of the schizothoracine fishes (Cyprinidae) revealed by the mitochondrial genome analyses. Gene.

[88] Lv F., Yang W.Y., Chen Z.T., Xu Q., Zhou Y.J., Du Y.Z. (2017). Three partial mitochondrial genomes from *Ips* (Coleoptera: Cruculionidae, Scolytinae) contribute to the phylogeny of Scolytinae. J. Asia-Pac. Enotomol..

[89] Wolfe J.A. (1994). An analysis of Neogene climates in Beringia. Palaeogeogr. Palaeoclimatol. Palaeoecol..

[90] Lanier G.N., Burkholder W.E., Birch M.C. (1974). Pheromones in speciation of Coleoptera. Pheromones.

[91] Mckenna D.D., Shin S., Ahrens D., Balke M., Beza-Beza C., Clarke D.J., Donath A., Escalona H.E., Friedrich F., Letsch H. (2019). The evolution and genomic basis of beetle diversity. Proc. Natl. Acad. Sci. USA.

[92] Meseguer A.S., Coeur A., Genson G., Jousselin E. Unravelling the historical biogeography and diversifification dynamics of a highly diverse conifer-feeding aphid genus. J. Biogeogr..

[93] Godefroid M., Meseguer A.S., Saune L., Genson G., Streito J., Rossi J., Riveron A.Z., Mayer F., Cruaud A., Rasplus J. (2019). Restriction-site associated DNA markers provide new insights into the evolutionary history of the bark beetle genus *Dendroctonus*. Mol. Phylogenet. Evol..

