# Supplementary material for: Insights into the Divergence of Chinese Ips Bark Beetles during Evolutionary Adaptation
Source: Biology (Basel). 2022 Feb 28;11(3):384. doi: 10.3390/biology11030384 (PMC8945085; doi:10.3390/biology11030384)
Supplement: Supplementary file 1 [file biology-11-00384-s001.zip › biology-1595414-supplementary.pdf]

Supplementary material

Table S1. The list of sample informaton of mitchondrial genomes of 26 published species of Scolytidae.

Subfamily	Tribe	Category	Species	Accession no.
Hylesininae	Hylastini	*Hylates*	*Hylates attenuatus*	NC_036290.1
Hylesininae	Hylastini	*Hylates*	*Hylates brunneus*	NC_036262.1
Ipinae	Ipini	*Pityogenes*	*Pityogenes trepanatus*	KX_035225.1
Ipinae	Ipini	*Pityogenes*	*Pityogenes bidentatus*	NC_036289.1
Ipinae	Ipini	*Ips*	*Ips calligraphus*	MW_589547
Ipinae	Ipini	*Ips*	*Ips acuminatus*	MK_988441
Ipinae	Ipini	*Ips*	*Ips sexdentatus*	NC_036281.1
Ipinae	Ipini	*Orthotomicus*	*Orthotomicus erosus*	MZ_823388
Ipinae	Ipini	*Orthotomicus*	*Orthotomicus laricis*	NC_036291.1
Ipinae	Dryocoetini	*Dryocoetes*	*Dryocoetes autographus*	NC_036287.1
Ipinae	Dryocoetini	*Dryocoetes*	*Dryocoetes hectographus*	MZ_766132
Ipinae	Dryocoetini	*Dryocoetes*	*Dryocoetes villosus*	NC_036282.1
Ipinae	Xyleborini	*Cyclorhipidion*	*Cyclorhipidion bodoanus*	NC_036295
Ipinae	Xyleborini	*Xylosandrus*	*Xylosandrus saxesenii*	KX035203.1
Ipinae	Xyleborini	*Anisandrus*	*Anisandrus dispar*	NC_036293.1
Ipinae	Xyleborini	*Xylosandrus*	*Xylosandrus morigerus*	NC_036283.1
Ipinae	Xyleborini	*Xylosandrus*	*Xylosandrus germanus*	NC_036280.1
Ipinae	Xyleborini	*Xylosandrus*	*Xylosandrus crassiusculus*	NC_036284.1
Ipinae	Xyloterini	*Trypodendron*	*Trypodendron signatum*	NC_036292.1
Ipinae	Xyloterini	*Trypodendron*	*Trypodendron domesticum*	NC_036286.1
Ipinae	Polygraphini	*Polygraphus*	*Polygraphus poligraphus*	OK_110248
Ipinae	Tryphphloeini	*Trypophloeus*	*Trypophloeus asperatus*	NC_036285.1
Ipinae	Corthylini	*Gnathotrchus*	*Gnathotrchus materiarius*	NC_036294.1
Ipinae	Corthylini	*Pityophthorus*	*Pityophthorus pubescens*	NC_036288.1
Ipinae	Hyloterini	*Dendroctonus*	*Dendroctonus micans*	MZ_768861
		Curculio	*Curculio elephas*	KX_087269.1

Table S2. *Ips* species used in this study.

species	Collection locality,data and collector	Host	Preservation method	Genbank Accession No.
Ips acuminatus (Gyllenhal)	Czech Republic: Moravia, Tulasice V-1995. M. Knizek	Unrecorded	Frozen	AF113325
Ips amitinus 1 (Eichhoff)	Czech Republic: Moravia, Chuchelna V-1995. M. Knizek Picea abies Ethanol AF113326	*Picea abies*	Ethanol	AF113326
Ips amitinus 2	Russia: Lenbolovo, St. Petersburg Reg. 8-10-VIII-1998.M. Mandelstan	Spruce	Ethanol	AF113327
Ips apache 1 (Lanier)	AZ: Cochise Co. Coronado National Forest. Rd 42, 10 mi NW of Portal. 6-IX-1996.	*Pinus engelmanni*	Frozen	AF113328
Ips apache 2	Mexico: Michoacan: Uruapan. Barranca del Cupatitzio 1996. A. Storer	Unrecorded	Ethanol	AF113329
Ips avulsus 1 (Eichhoff)	Louisiana. 1996. G. Lenhard	Unrecorded	Pinned	AF113330
Ips avulsus 2	GA: Cobb Co. Marietta. 19-IX-1997. M. Caterino	*Pinus*	Frozen	AF113331
Ips bonanseai 1 (Hopkins)	AZ: Graham Co. Coronado National Forest Rd. 366.5-IX-1996.	*Pinus*	Frozen	AF113332
Ips bonanseai	2 Mexico: Nuevo Leon. XII-1993. S. Seybold	Unrecorded	Frozen	AF113333
Ips borealis Swaine	Canada: Quebec, Gatineau Park nr. Tayor Lake.20-V-1995.	*Picea glauca*	Frozen	AF113334
Ips calligraphus 1 (Germar)	NY: Suffolk Co. Smithtown 11-IX-1994.	*Pinus strobus*	Frozen	AF113335
Ips calligraphus 2	AZ: Gila Co. 14 mi. S. Young 26-XI-1985. G. N. Lanier	*Pinus ponderosa*	Pinned	AF113336
Ips cembrae 1 (Heer)	Czech Republic: Moravia, Resice. 15-V-1997. M. Knizek	*Larix decidua*	Ethanol	AF113337
Ips cembrae 2	France: Brianc¸on. 16-VI-1996. A. Roques	*Larix decidua*	Ethanol	AF113338
Ips cembrae 3	China: Heilongjiang, Lo-Shan. 1-VI-1996. A. Roques	*Larix gmelini*	Ethanol	AF113339
Ips concinnus (Mannerheim)	WA: Clallam Co. Hoh River Rd. 28-VI-1997.	*Picea sitchensis*	Frozen	AF113340
Ips confusus 1 (LeConte)	AZ: Gila Co. San Carlos Indian Reservation. 11 mi. NE of Point of Pines 26-V-1993. S. Seybold, A. I. Cognato & D. L. Wood	*Pinus edulis*	Frozen	AF113341
Ips confusus 2	NV: Douglas Co. Gardenerville. II-1994. S. Seybold.	*Pinus monophylla*	Frozen	AF113342
Ips cribricollis 1 (Eichhoff)	NM: Otero Co. Lincoln National Forest Rt. 82 8.05 km E. Cloudcroft, 11-V-1994.E.	*Pinus ponderosa*	Frozen	AF113343
Ips cribricollis 2	Mexico: Michoacan: San Juan Nuevo, LaPila Forest,31-V-1998. A. I. Cognato & A. Del Rio-Morea	*Pinus leiophylla*	Ethanol	AF113344
Ips duplicatus 1 (Sahlberg)	Czech Republic: Moravia, Chuchelna. V-1995. M.Knizek	*Picea abies*	Ethanol	AF113345
Ips duplicatus 2	Russia: Lenbolovo, St. Petersburg Reg. 8-10-VIII-1998.M. Mandelstan	Spruce	Ethanol	AF113346
Ips emarginatus 1	CA: Lassen Co. Black's Mountain Experimental Forest.VII-1994. S.	*Pinus*	Frozen	AF113347

(LeConte)	Seybold, A. I. Cognato & D. L. Wood	*ponderosa*		
Ips emarginatus 2	WA: Kittitas Co. Roslyn. 30-VI-1997.	*Pinus ponderosa*	Frozen	AF113348
Ips grandicollis 1 (Eichhoff)	NY: Suffolk Co. Brookhaven. 28-VIII-1992	*Pinus rigida*	Frozen	AF113349
Ips grandicollis 2	Canada: Ontario: Constance Bay, Turbolton Forest.13-V-1995.	*Pinus banksiana*	Pinned	AF113350
Ips grandicollis 3	FL: Pinellas Co., Harpon Spring, Howard Park.12-V-1995. D. Czokajlo	Unrecorded	Pinned	AF113351
Ips hauseri Reitter	USSR: Kazachstan. Isyk 1767m Zailij. Ala Tau.29-V-1974. A. Pfeffer Igt.	Unrecorded	Pinned	AF113352
Ips hoppingi 1 Lanier	Mexico: Nuevo Leon Mpio. Galeana Rd. Galeana-Dr.Arroyo, Las Crucites 2,470m. X-1993. S. Seybold	*Pinus cembroides*	Frozen	AF113353
Ips hoppingi 2	AZ: Cochise Co. Coronado N.F. Rd. 42 10mi NW. Portal.7-IX-1996.	*Pinus cembroides*	Frozen	AF113354
Ips hunteri Swaine	AZ: Greenlee Co. Rd. 191, 3 mi. N. Hannangan's Meadow. 3-IX-1996.	*Picea pugens*	Frozen	AF113355
Ips integer (Eichhoff)	AZ: Greenlee Co. Rd. 191, 7 mi. S. Hannangan's Meadow. 1-IX-1996.	*Pinus ponderosa*	Frozen	AF113356
Ips knausi Swaine	CO: Fairfeild nr. Payosa Springs N. Jose. VII-1994. S.Kelley	*Pinus ponderosa*	Ethanol	AF113357
Ips latidens 1 (Leconte)	CA: Mariposa Co. Fish Camp 15-IV-1996.	*Pinus lambertinan*	Frozen	AF113358
Ips latidens 2	WA: Roslyn. 30-VI-1997.	*Pinus ponderosa*	Frozen	AF113359
Ips lecontei 1 Swaine	AZ: Gila Co. San Carlos Indian Reservation. Point of Pines 27-V-1993. S. Seybold, A. I. Cognato & D. L. Wood	*Pinus ponderosa*	Frozen	AF113360
Ips lecontei 2	AZ: Greenlee Co. Apache National Forest Rd. 191, 20 mi N.Morceani. 4-IX-1996.	*Pinus ponderosa*	Frozen	AF113361
Ips longifolia (Stebbing)	Bhutan: Autsu Lhuntshi. 1100 m. 9-X-1985. H. Schmutzenhofer	Chir pine	Pinned	AF113362
Ips mannsfeldi (Wachtl)	Austria: Vienna. VI-1989. C. Stauffer	Unrecorded	Ethanol	AF113363
Ips mexicanus (Hopkins)	CA: Marin Co. Olema. 8-III.1994.	*Pinus muricata*	Frozen	AF113364
Ips montanus (Eichhoff)	CA: Modoc Co. Warner Mountains, T44N, R15E, N1/2, S17.25-VII-1994. S. Seybold, A. I. Cognato, D. L. Wood.	*Pinus monticolla*	Frozen	AF113365
Ips nobilis (Wollaston)	Canary Islands: Tenerife. Villaflor. 12-III-1993. M. Knizek.	Unrecorded	Pinned	AF113366
Ips paraconfusus 1 Lanier	CA: Alameda Co. Berkeley. Wildcat Cayon Rd. 14-X-1995.	*Pinus radiata*	Frozen	AF113367
Ips paraconfusus 2 Lanier	CA: Mariposa Co. Fish Camp 15-IV-1996.	*Pinus lambertinana*	Frozen	AF113368
Ips perroti Swaine	Canada: Ontario: Constance Bay, Torbolton Forest. 13-V-1995	*Pinus bankisana*	Frozen	AF113369

Ips perturbatus 1(Eichhoff)	Canada: Ontario: Muni. of Ottawa-Carton, MarlboroughForest, 19-V-1995. D. E. Bright & A. I. Cognato	*Picea glauca*	Frozen	AF113370
Ips perturbatus 2, 3	Canada: Alberta. nr Manning. VIII-1996. D. Langor	*Picea glauca*	Ethanol	AF113371
Ips perturbatus 2, 3	Canada: Alberta. nr Manning. VIII-1996. D. Langor	*Picea glauca*	Ethanol	AF113372
Ips pilifrons 1 Swaine	AZ: Greenlee Co. Rd. 191, 7 mi. S. Hannangan's Meadow.1-IX-1996	*Picea engelmannii*	Frozen	AF113373
Ips pilifrons 2	CO: Larmie Co. Roosevelt N.F., Hwy 14 10,000 ft. 18-VI-1997	*Picea engelmannii*	Frozen	AF113374
Ips pini 1 (Say)	NM: Otero Co. Lincoln N.F. 11-V-1994.	*Pinus ponderosa*	Frozen	AF113375
Ips pini 2	AZ: Apache Co. Apache N.F. Rd. 56. 3-IX-1996.	*Pinus ponderosa*	Frozen	AF113376
Ips pini 3	Canada: Calgary 9-X-1990. J. Bordan	*Pinus contorta*	Ethanol	AF113377
Ips plastographus (LeConte)	CA: Marin Co. Inverness 14-VIII-1995.	*Pinus muricata*	Frozen	AF113378
Ips schmutzenhoferi Holzschuh	Bhutan: Chelaila, 3100 m. 5-VI-1986. H. Schmutzenhofer	*Larix griffithiana*	Pinned	AF113379
Ips sexdentatus (Boerner)	Czech Republic: Moravia, Tulasice V-1995. M. Knizek	Unrecorded	Ethanol	AF113380
Ips spinifer (Eichhoff)	CA: Contra Costa Co. Diablo 3-IX-1995.	*Pinus sabiniana*	Frozen	AF113381
Ips stebbingi 1 Strohmeyer	Pakistan: Swat, Utror. 3-VIII-1987. C. Holzschuh	*Pinus gerardiana*	Pinned	AF113382
Ips stebbingi	2 Nepal: Dhawalagiri, Kall-Gandaki-Khola, Mustang, D.Kalopani 2500-2800 m 3-VIII-1987. C. Holzschuh	*Pinus gerardiana*	Pinned	AF113383
Ips tridens(Mannerheim)	WA: Clallam Co. Hoh River Rd. 28-VI-1997.	*Picea sitchensis*	Frozen	AF113384
Ips typographus 1(Linnaeus)	Czech Republic: Moravia. V-1995. M. Knizek	Unrecorded	Frozen	AF113385
Ips typographus 2	Russia: Lenbolovo, St. Petersburg Reg. 8-10-VIII-1998. M.Mandelstan	Spruce	Ethanol	AF113386
Ips typographus 3	Russia: St. Khekhcir evirons, Khabarousk 2-IX-1990. M.Mandelstan	*Pinus koraiensis*	Pinned	AF113387
Ips woodi 1 Thatcher	AZ: Greenlee Co. Rd. 191, 7 mi. S. Hannangan's Meadow. 1-IX-1996.	*Pinus strobiformis*	Frozen	AF113388
Ips woodi 2	CA: Mono Co. Inyo National Forest Crooked Creek Research Station 21-VIII-1997.	*Pinus flexilis*	Frozen	AF113389
Ips amitinus	Lower Austria, Gutenbrunn	*Picea abies*	Ethanol	U82587
Ips cembrae	Styria, Kindberg	*Larix decidua*	Ethanol	U82588
Ips duplicatus	Morawia, Ostrau	*Picea abies*	Ethanol	U82586
Ips acuminatus	Lower Austria, Retz	*Pinus*	Ethanol	U82585

		silvestris		
Ips subelongatus	China: Hebei,Pingquan	*Larix principis*	Ethanol	KC411926
Ips subelongatus	China: Neimenggu,Aertai,Wuchagou	*Larix gmelinii*	Ethanol	KC411927
Ips subelongatus	China: Heilongjiang,Menjiagang	*Larix olgensis*	Ethanol	KC411928
Ips typographus	Belgium Arlon	Spruce	Ethanol	KF846139
Ips typographus	Belgium Beauraing	Spruce	Ethanol	KF846140
Ips typographus	Belgium Bouillon	Spruce	Ethanol	KF846141
Ips hoppingi	Unrecorded	*Pinus*	Ethanol	EF115516
Ips hunteri	Unrecorded	Spruce	Ethanol	EF115517
Ips amitinus	Unrecorded	Spruce	Ethanol	EF115509
Ips perturbatus	Unrecorded	Spruce	Ethanol	EF115521
Ips pilifrons	Unrecorded	Spruce	Ethanol	EF115523
Ips shangrila	Unrecorded	Spruce	Ethanol	EF115526
Ips tridens	Unrecorded	Spruce	Ethanol	EF115528
Ips cembrae	Unrecorded	*Larix*	Ethanol	KC514452
Ips cembrae	Unrecorded	*Larix*	Ethanol	KC514451
Ips nitidus	Unrecorded	Spruce	Ethanol	KF846204
Ips nitidus2	Unrecorded	Spruce	Ethanol	KF846205

Table S3. Base composition in the mitochondrial genomes of bark beetles.

Whole genome	Length (bp)	T %	A %	A+T %	AT-skew	C %	G %	C+G %	GC-skew
Dendroctonus micans	14,607	34.15	40.55	74.70	0.09	15.93	9.37	25.30	-0.26
Ips nitidus	15,409	35.16	37.97	73.13	0.04	16.65	10.22	26.87	-0.24
Ips shangrila	15,641	35.15	38.11	73.25	0.04	16.57	10.18	26.75	-0.24
Ips hauseri	15,466	34.83	37.61	72.43	0.04	17.42	10.15	27.57	-0.26
Ips duplicatus	15,572	34.59	37.30	71.89	0.04	17.74	10.36	28.11	-0.26
Ips subelongatus	15,259	35.76	38.76	74.52	0.04	15.73	9.75	25.48	-0.23
Ips typographus	15,384	35.22	38.22	73.44	0.04	16.63	9.93	26.56	-0.25
Average(*Ips*)		35.12	37.99	73.11	0.04	16.79	10.10	26.89	-0.25
Protein coding genes(PCGs)									
Dendroctonus micans	11,126	41.77	31.57	73.34	-0.14	14.38	12.28	26.66	-0.08
Ips nitidus	11,190	41.39	29.71	71.09	-0.16	15.40	13.51	28.91	-0.07
Ips shangrila	11,160	41.47	30.07	71.54	-0.16	15.16	13.29	28.46	-0.07
Ips hauseri	11,177	41.07	29.13	70.20	-0.17	15.96	13.84	29.80	-0.07
Ips duplicatus	11,137	40.59	29.20	69.79	-0.16	16.38	13.83	30.21	-0.08
Ips subelongatus	11,196	42.42	30.46	72.88	-0.16	14.19	12.92	27.12	-0.05
Ips typographus	11,196	41.44	30.01	71.45	-0.16	15.26	13.28	28.55	-0.07
Average(*Ips*)		41.40	29.76	71.16	-0.16	15.39	13.45	28.84	-0.07
rRNA									
Dendroctonus micans	2,137	43.61	36.17	79.78	-0.09	6.69	13.52	20.22	0.34
Ips nitidus	2,181	40.15	38.23	78.38	-0.02	7.07	14.55	21.62	0.35
Ips shangrila	2,201	40.80	37.30	78.10	-0.04	6.93	14.97	21.90	0.37
Ips hauseri	2,159	40.10	38.61	78.71	-0.02	6.69	14.60	21.29	0.37
Ips duplicatus	2,203	39.96	38.75	78.71	-0.02	6.64	14.65	21.29	0.38
Ips subelongatus	2,177	40.57	38.37	78.94	-0.03	6.50	14.55	21.06	0.38
Ips typographus	2,177	40.43	37.86	78.29	-0.03	6.88	14.83	21.71	0.37
Average(*Ips*)		40.34	38.18	78.52	-0.03	6.79	14.69	21.48	0.37
tRNA									
Dendroctonus micans	1,358	37.78	39.54	77.32	0.02	10.31	12.37	22.68	0.09
Ips nitidus	1,444	36.23	40.35	76.58	0.05	10.24	13.18	23.42	0.13
Ips shangrila	1,444	36.52	40.21	76.73	0.05	10.82	12.44	23.27	0.07
Ips hauseri	1,438	36.89	38.95	75.85	0.03	10.16	13.99	24.15	0.16
Ips duplicatus	1,429	36.52	39.40	75.92	0.04	10.38	13.70	24.08	0.14
Ips subelongatus	1,431	37.26	40.43	77.69	0.04	9.94	12.37	22.31	0.11
Ips typographus	1,439	35.57	40.43	75.99	0.06	10.90	13.11	24.01	0.09
Average(*Ips*)		36.50	39.96	76.46	0.05	10.41	13.13	23.54	0.12

Table S4. Origanization of the mitochondrial genome of *I. typographus*.

Gene	Direction	Start position	Stop position	Length	Anticodon	Start codon	Stop codon	IGS
trnQ	R	1	71	71	CAT			
trnM	F	86	157	72	TTG			14
ND2	F	161	1168	1008		ATA	TAA	3
trnW	F	1169	1236	68	TCA			0
trnC	R	1266	1332	67	GCA			29
trnY	R	1338	1404	67	GTA			5
COI	F	1429	2997	1569		ATT	TAA	24
trnL2	F	3021	3089	69	TAA			23
COII	F	3090	3770	681		ATC	TAA	0
trnK	F	3805	3876	72	CTT			34
trnD	F	3893	3960	68	GTC			16
ATP8	F	3961	4125	165		ATC	TAG	0
ATP6	F	4122	4799	678		ATA	TAA	-4
COIII	F	4805	5587	783		ATG	TAA	5
trnG	F	5614	5679	66	TCC			26
ND3	F	5683	6033	351		ATT	TAA	3
trnA	F	6046	6114	69	TGC			12
trnR	F	6136	6205	70	TCG			21
trnN	F	6238	6303	66	GTT			32
trnS1	F	6304	6371	68	GCT			0
trnE	F	6373	6443	71	TTC			1
trnF	R	6448	6518	71	GAA			4
ND5	R	6552	8297	1746		ATT	TAA	33
trnH	R	8298	8363	66	GTG			0
ND4	R	8398	9735	1338		ATG	TAA	34
ND4L	R	9742	10029	288		ATT	TAA	6
trnT	F	10055	10121	67	TGT			25
trnP	R	10122	10188	67	TGG			0
ND6	F	10200	10697	498		ATT	TAA	11
CytB	F	10783	11925	1143		ATA	TAA	85
trnS2	F	12028	12097	70	TGA			102
ND1	R	12117	13064	948		TTG	TAA	19
trnL1	R	13066	13134	69	TAG			1
rrnL	R	13152	14516	1365				17
trnV	R	14510	14574	65	TAC			-7
rrnS	R	14573	15384	812				-2

Table S5. Origianization of the mitochondrial genome of *I. subelongatus*.

Gene	Direction	Start position	Stop position	Length	Anticodon	Start codon	Stop codon	IGS
trnQ	R	1	71	71	TTG			
trnM	F	140	210	71	CAT			68
ND2	F	214	1221	1008		ATA	TAA	3
trnW	F	1223	1289	67	TCA			1
trnC	R	1296	1359	64	GCA			6
trnY	R	1373	1440	68	GTA			13
COI	F	1452	2999	1548		ATT	TAA	11
trnL2	F	3012	3079	68	TAA			12
COII	F	3080	3760	681		ATC	TAA	0
trnK	F	3804	3874	71	CTT			43
trnD	F	3880	3947	68	GTC			5
ATP8	F	3948	4121	174		ATT	TAG	0
ATP6	F	4115	4795	681		ATG	TAA	-7
COIII	F	4820	5602	783		ATG	TAA	24
trnG	F	5630	5700	71	TCC			27
ND3	F	5704	6054	351		ATC	TAA	3
trnA	F	6064	6128	65	TGC			9
trnR	F	6147	6215	69	TCG			18
trnN	F	6248	6314	67	GTT			32
trnS1	F	6315	6381	67	GCT			0
trnE	F	6384	6448	65	TTC			2
trnF	R	6456	6524	69	GAA			7
ND5	R	6534	8276	1743		ATT	TAA	9
trnH	R	8277	8348	72	GTG			0
ND4	R	8373	9710	1338		ATG	TAA	24
ND4L	R	9726	10019	294		ATA	TAA	15
trnT	F	10067	10133	67	TGT			47
trnP	R	10134	10199	66	TGG			0
ND6	F	10202	10711	510		ATT	TAA	2
CytB	F	10761	11897	1137		ATG	TAA	49
trnS2	F	11912	11981	70	TGA			14
ND1	R	12001	12948	948		TTG	TAA	19
trnL1	R	12950	13018	69	TAG			1
rrnL	R	13004	14367	1364				-15
trnV	R	14383	14448	66	TAC			15
rrnS	R	14447	15259	813				-2

Table S6. Origanization of the mitochondrial genome of *I. shangrila.*

Gene	Direction	Start position	Stop position	Length	Anticodon	Start codon	Stop codon	IGS
trnQ	R	1	70	70	TTG			
trnM	F	101	170	70	CAT			30
ND2	F	171	1181	1011		ATT	TAA	0
trnW	F	1197	1265	69	TCA			15
trnC	R	1344	1410	67	GCA			78
trnY	R	1463	1528	66	GTA			52
COI	F	1595	3139	1545		TTA	TAA	66
trnL2	F	3150	3216	67	TAA			10
COII	F	3217	3897	681		ATC	TAA	0
trnK	F	3929	4000	72	CTT			31
trnD	F	4012	4080	69	GTC			11
ATP8	F	4081	4248	168		ATT	TAG	0
ATP6	F	4245	4922	678		ATA	TAA	-4
COIII	F	4933	5715	783		ATG	TAA	10
trnG	F	5740	5813	74	TCC			24
ND3	F	5817	6167	351		ATT	TAA	3
trnA	F	6173	6241	69	TGC			5
trnR	F	6303	6372	70	TCG			61
trnN	F	6413	6478	66	GTT			40
trnS1	F	6479	6548	70	GCT			0
trnE	F	6551	6618	68	TTC			2
trnF	R	6623	6687	65	GAA			4
ND5	R	6704	8440	1737		ATT	TAA	16
trnH	R	8441	8509	69	GTG			0
ND4	R	8543	9880	1338		ATG	TAA	33
ND4L	R	9899	10192	294		ATA	TAA	18
trnT	F	10253	10320	68	TGT			60
trnP	R	10321	10387	67	TGG			0
ND6	F	10402	10896	495		ATT	TAA	14
CytB	F	11062	12195	1134		ATG	TAA	165
trnS2	F	12288	12360	73	TGA			92
ND1	R	12382	13326	945		ATT	TAA	21
trnL1	R	13331	13397	67	TAG			4
rrnL	R	13383	14766	1384				-15
trnV	R	14760	14827	68	TAC			-7
rrnS	R	14825	15641	817				-3

Table S7. Origanization of the mitochondrial genome of *I. duplicatus.*

Gene	Direction	Start position	Stop position	Length	Anticodon	Start codon	Stop codon	IGS
trnQ	R	1	69	69	TTG			
trnM	F	135	203	69	CAT			65
ND2	F	204	1214	1011		ATA	TAA	0
trnW	F	1240	1306	67	TCA			25
trnC	R	1324	1387	64	GCA			17
trnY	R	1477	1547	71	GTA			89
COI	F	1562	3106	1545		ATC	TAA	14
trnL2	F	3102	3169	68	TAA			-5
COII	F	3170	3850	681		ATT	TAA	0
trnK	F	3902	3972	71	CTT			51
trnD	F	4026	4094	69	GTC			53
ATP8	F	4095	4268	174		ATT	TAG	0
ATP6	F	4262	4942	681		ATG	TAA	-7
COIII	F	4952	5734	783		ATG	TAA	9
trnG	F	5816	5886	71	TCC			81
ND3	F	5887	6240	354		ATT	TAA	0
trnA	F	6251	6316	66	TGC			10
trnR	F	6331	6399	69	TCG			14
trnN	F	6434	6499	66	GTT			34
trnS1	F	6500	6567	68	GCT			0
trnE	F	6570	6635	66	TTC			2
trnF	R	6711	6779	69	GAA			75
ND5	R	6834	8541	1708		ATC	T-	54
trnH	R	8542	8611	70	GTG			0
ND4	R	8624	9961	1338		ATG	TAG	12
ND4L	R	9974	10267	294		ATA	TAG	12
trnT	F	10285	10351	67	TGT			17
trnP	R	10352	10418	67	TGG			0
ND6	F	10442	10927	486		ATC	TAA	23
CytB	F	10983	12122	1140		ATG	TAA	55
trnS2	F	12231	12299	69	TGA			108
ND1	R	12327	13268	942		ATT	TAA	27
trnL1	R	13273	13341	69	TAG			4
rrnL	R	13317	14717	1401				-25
trnV	R	14706	14772	67	TAC			-12
rrnS	R	14771	15572	802				-2

Table S8. Origianization of the mitochondrial genome of *I. hauseri.*

Gene	Direction	Start position	Stop position	Length	Anticodon	Start codon	Stop codon	IGS
trnQ	R	1	70	70	TTG			
trnM	F	123	193	71	CAT			52
ND2	F	194	1204	1011		ATA	TAA	0
trnW	F	1226	1292	67	TCA			21
trnC	R	1309	1373	65	GCA			16
trnY	R	1439	1506	68	GTA			65
COI	F	1470	3050	1581		ATT	TAA	-37
trnL2	F	3046	3113	68	TAA			-5
COII	F	3114	3794	681		ATT	TAA	0
trnK	F	3835	3905	71	CTT			40
trnD	F	3951	4019	69	GTC			45
ATP8	F	4020	4190	171		ATC	TAG	0
ATP6	F	4184	4864	681		ATG	TAA	-7
COIII	F	4876	5658	783		ATG	TAA	11
trnG	F	5714	5783	70	TCC			55
ND3	F	5784	6137	354		ATT	TAA	0
trnA	F	6150	6218	69	TGC			12
trnR	F	6317	6386	70	TCG			98
trnN	F	6418	6483	66	GTT			31
trnS1	F	6484	6551	6	GCT			0
trnE	F	6554	6621	68	TTC			2
trnF	R	6656	6723	68	GAA			34
ND5	R	6781	8488	1708		ATC	T-	57
trnH	R	8489	8557	69	GTG			0
ND4	R	8570	9907	1338		ATG	TAA	12
ND4L	R	9921	10214	294		ATA	TAG	13
trnT	F	10231	10297	67	TGT			16
trnP	R	10298	10365	68	TGG			0
ND6	F	10377	10874	498		ATT	TAA	11
CytB	F	10924	12063	1140		ATG	TAA	49
trnS2	F	12172	12240	69	TGA			108
ND1	R	12276	13212	937		TTG	T	35
trnL1	R	13214	13283	70	TAG			1
rrnL	R	13300	14660	1361				16
trnV	R	14654	14720	67	TAC			-7
rrnS	R	14719	15516	798				-2

Table S9. Origanization of the mitochondrial genome of *I. nitidus*.

Gene	Direction	Start position	Stop position	Length	Anticodon	Start codon	Stop codon	IGS
trnQ	R	1	71	71	TTG			
trnM	F	87	159	73	CAT			15
ND2	F	163	1170	1008		ATA	TAA	3
trnW	F	1171	1238	68	TCA			0
trnC	R	1264	1333	70	GCA			25
trnY	R	1340	1406	67	GTA			6
COI	F	1444	3003	1560		ATC	TAA	37
trnL2	F	3023	3091	69	TAA			19
COII	F	3092	3772	681		ATC	TAA	0
trnK	F	3803	3874	72	CTT			30
trnD	F	3891	3958	68	GTC			16
ATP8	F	3959	4123	165		ATT	TAG	0
ATP6	F	4120	4797	678		ATA	TAA	-4
COIII	F	4803	5585	783		ATG	TAA	5
trnG	F	5611	5676	66	TCC			25
ND3	F	5677	6030	354		ATT	TAA	0
trnA	F	6042	6110	69	TGC			11
trnR	F	6132	6201	70	TCG			21
trnN	F	6234	6299	66	GTT			32
trnS1	F	6300	6367	68	GCT			0
trnE	F	6369	6440	72	TTC			1
trnF	R	6445	6516	72	GAA			4
ND5	R	6542	8287	1746		ATT	TAA	25
trnH	R	8288	8353	66	GTG			0
ND4	R	8393	9730	1338		ATG	TAA	39
ND4L	R	9737	10024	288		ATT	TAA	6
trnT	F	10051	10117	67	TGT			26
trnP	R	10118	10184	67	TGG			0
ND6	F	10196	10693	498		ATT	TAA	11
CytB	F	10784	11926	1143		ATA	TAA	90
trnS2	F	12049	12118	70	TGA			122
ND1	R	12138	13085	948		TTG	TAA	19
trnL1	R	13087	13155	69	TAG			1
rrnL	R	13173	14536	1364				17
trnV	R	14530	14594	65	TAC			-7
rrnS	R	14593	15409	817				-2

Table S10. Origanization of the mitochondrial genome of *D. micans.*

Gene	Direction	Start position	Stop position	Length	Anticodon	Start codon	Stop codon	IGS
trnQ	R	1	70	70	TTG			
trnM	F	81	150	70	CAT			10
ND2	F	151	1150	1000		ATT	T	0
trnW	F	1172	1235	64	TCA			21
trnC	R	1235	1296	62	GCA			-1
trnY	R	1300	1362	63	GTA			3
COI	F	1355	2899	1545		ATT	TAA	-8
trnL2	F	2895	2959	65	TAA			-5
COII	F	2960	3643	684		ATT	TAA	0
trnK	F	3645	3715	71	CTT			1
trnD	F	3716	3779	64	GTC			0
ATP8	F	3780	3935	156		ATA	TAG	0
ATP6	F	3982	4600	619		ATA	TAA	46
COIII	F	4606	5388	783		ATG	TAA	5
trnG	F	5395	5458	64	TCC			6
ND3	F	5459	5812	354		ATT	TAG	0
trnA	F	5811	5871	61	TGC			-2
trnR	F	5870	5936	67	TCG			-2
trnN	F	5935	6000	66	GTT			-2
trnS1	F	6001	6065	65	GCT			0
trnE	F	6065	6127	63	TTC			-1
trnF	R	6126	6188	63	GAA			-2
ND5	R	6192	7896	1705		ATT	T	3
trnH	R	7897	7957	61	GTG			0
ND4	R	7941	9284	1344		ATA	TAA	-17
ND4L	R	9281	9577	297		ATG	TAA	-4
trnT	F	9581	9643	63	TGT			3
trnP	R	9644	9706	63	TGG			0
ND6	F	9709	10212	504		ATT	TAA	2
CytB	F	10215	11351	1137		ATA	TAA	2
trnS2	F	11354	11418	65	TGA			2
ND1	R	11436	12383	948		TTG	TAG	17
trnL1	R	12385	12446	62	TAG			1
rrnL	R	12409	13742	1334				-38
trnV	R	13741	13806	66	TAC			-2
rrnS	R	13804	14607	804				-3

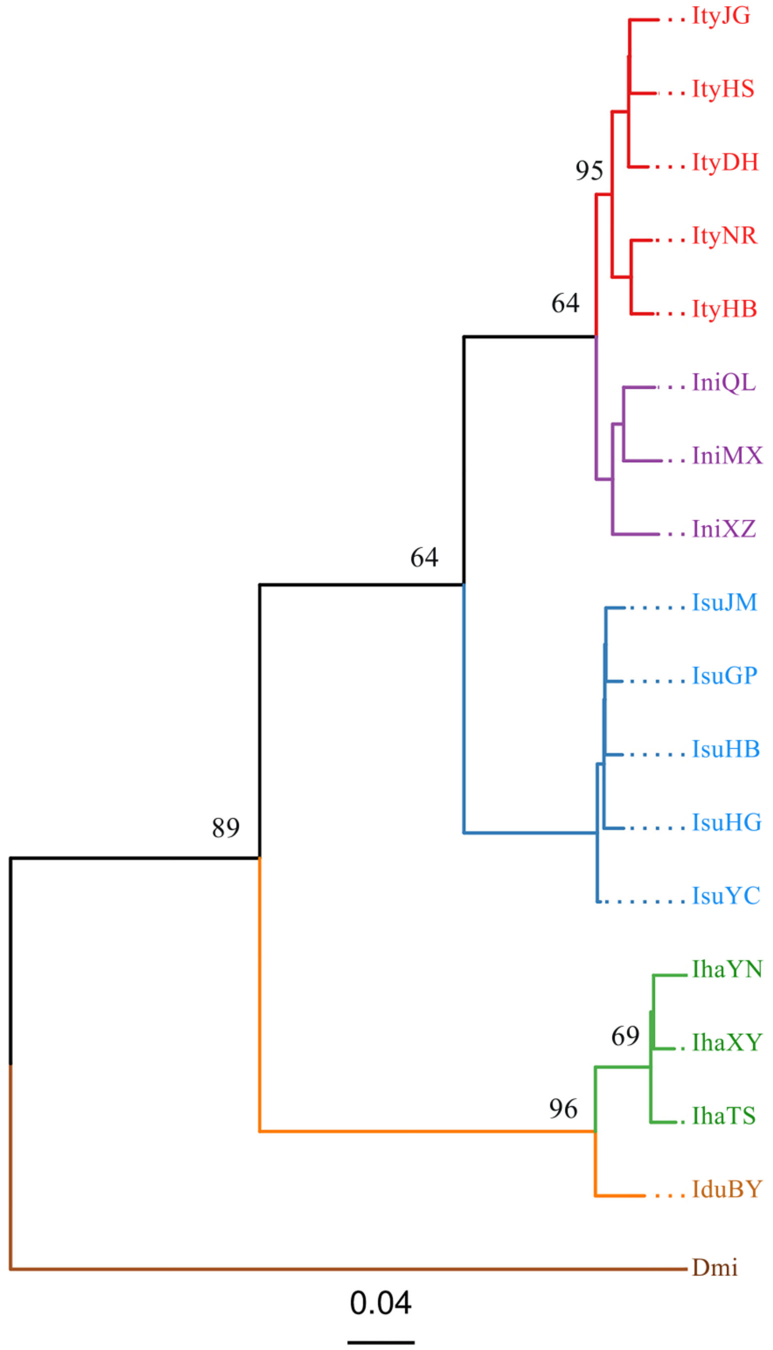

Figure S1. Phylogenetic tree (ML) of *Ips* geographic populations based on SNP. Numbers above or below branches indicate bootstrap values.

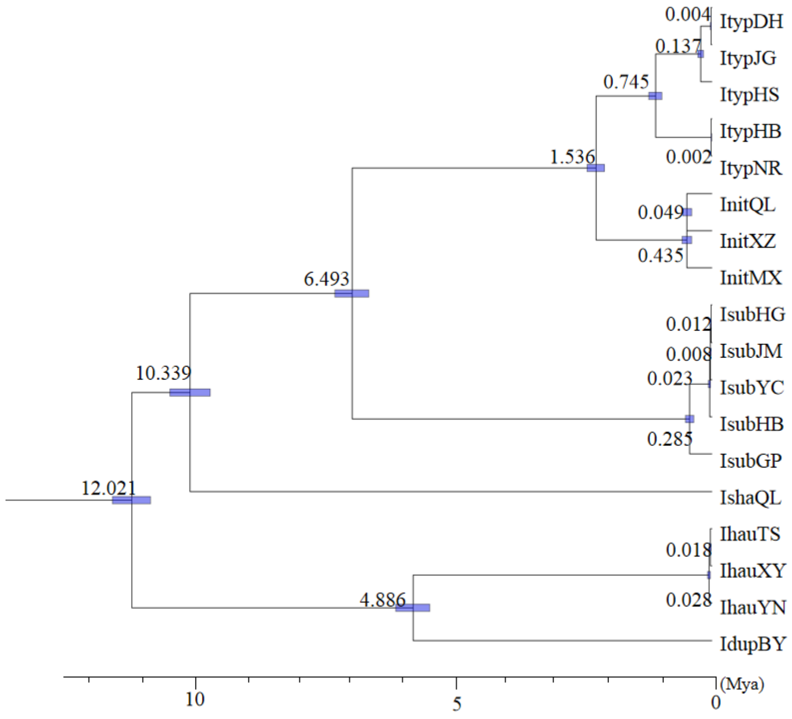

Figure S2. Tree of divergence times tree of the *Ips* species/populations.

Numbers above branches indicate the divergence time (in million years) of the branches. The middle solid line indicates the mean population size, and the gray area below and above the solid line indicates the 95% confidence interval.

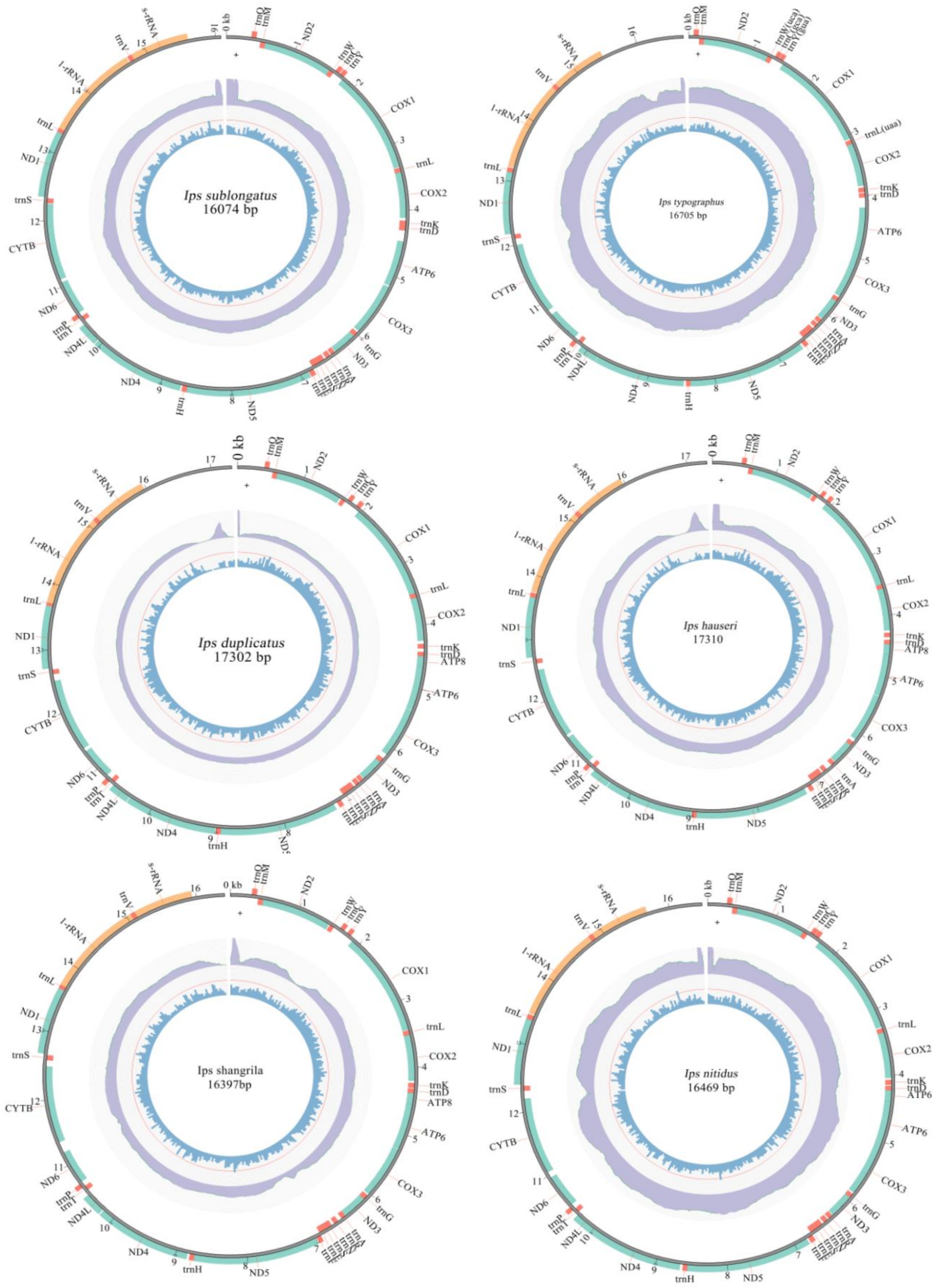

Figure S3. Circular maps of six newly sequenced bark beetles. The genes that were transcribed clockwise are shown outside the circle, while genes that were transcribed counterclockwise are shown inside the circle.

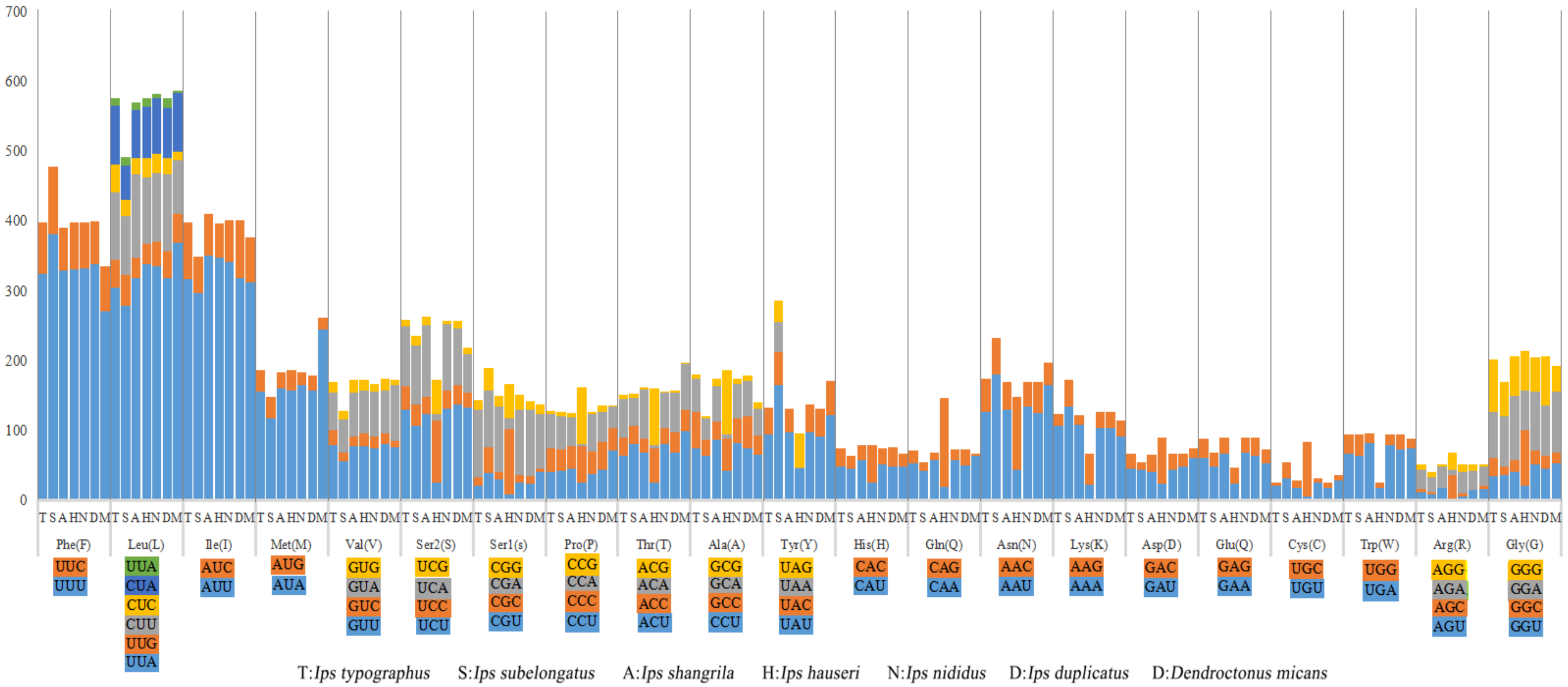

Figure S4. Amino acid usage of protein-coding genes of the mitochondrial genome in the genus *Ips*.
